# Exploration of marine bacterioplankton community assembly mechanisms during chemical dispersant and surfactant‐assisted oil biodegradation

**DOI:** 10.1002/ece3.8091

**Published:** 2021-09-10

**Authors:** Christina Nikolova, Umer Zeeshan Ijaz, Tony Gutierrez

**Affiliations:** ^1^ Institute of Mechanical, Process and Energy Engineering School of Engineering and Physical Sciences Heriot‐Watt University Edinburgh UK; ^2^ School of Engineering University of Glasgow Glasgow UK

**Keywords:** community assembly, crude oil, deterministic, dispersant, marine, microbial, null model

## Abstract

Assembly processes in marine microbial communities amended with crude oil and chemical dispersant are poorly understood and even more so when biosurfactants are used. We set up a microcosm experiment in which microbiome structure was analyzed using 16S rRNA gene amplicon sequencing and six null models to better understand and quantify the mechanisms and patterns controlling the assembly of a marine crude oil degrading microbial community in the presence of chemical dispersant or rhamnolipid biosurfactant. Although each null model quantifies different aspects of the community assembly, there was a general agreement that neither purely stochastic nor purely deterministic processes dominated the microbial communities, and their influence was variable over time. Determinism was dominant in the early phase of incubation, while stochasticity was prevalent in the middle and late stages. There was faster recruitment of phylogenetically distant species in the dispersant‐amended community compared to oil‐only or rhamnolipid‐amended communities. This analysis provides important insights of how chemical dispersants and rhamnolipid influence microbial communities' dynamics and identified which groups may be excluded—an important consideration for biodegradation process and oil spill response.

## Graphical abstract

Assembly processes in marine microbial communities amended with crude oil and chemical dispersant are poorly understood and even more so when biosurfactants are used. We used a comprehensive set of null models approaches to test traditional macroecological concepts in the context of microbial metagenomics in order to understand and quantify the mechanisms and patterns controlling the complexity of microbial ecology Our results advance the understanding of how chemical and biogenic dispersants affect the ecological processes in marine microbial community response to crude oil.

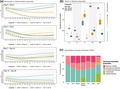

## 1 INTRODUCTION

Marine bacterioplankton play a vital role in dissolved organic carbon cycling, nutrient recycling, and photosynthesis and form the basis of the marine food web (Azam et al., 1983). Until the development of next‐generation sequencing (NGS), little was known about the diversity, functions, and community structures of marine bacteria (Amann et al., 1995). There is a general lack of understanding of how microbial communities assemble under varying conditions and environmental settings. Several recent studies have demonstrated that through improved methodology and interpretation, null models can provide valuable insight of the underlying ecological processes in microbial community assembly and in estimating their relative importance (Presley et al., 2010; Stegen et al., 2015a; Tucker et al., 2016; Vass et al., 2020; Verster & Borenstein, 2018). For instance, the assembly of rock pool (Vass et al., 2020) and anaerobic digestion (Trego et al., 2021) communities were assessed using a combination of null models, showing that dispersal limitation might have a more important role in the community assembly than previously thought. In a human gut microbiome, a lottery‐based null model identified a clade‐based assembly in which one group or species is the “winner” (hence the name “lottery”) that solely occupies a given niche due to a competitive advantage over other, often closely related, species (Verster & Borenstein, 2018). Furthermore, a null model developed by Darcy et al. (2020) revealed that human microbiome generally follows what the authors called the “nepotism” hypothesis, that is, close relatives are more likely to be recruited in a community than distant relatives (also known as phylogenetic underdispersion) in contrast to the lottery winner model. Tucker et al.'s framework (Tucker et al., 2016), which differentiates between neutral and niche community, has been applied to investigate the response of soil microbial communities to an anthropogenic disturbance (Lee et al., 2017) and to explore the role of ecological selection, drift, and dispersal in assembling the bacterial communities associated with domesticated and wild wheat species (Hassani et al., 2020).

Through a combination of several approaches and ecological frameworks (see Table 1), we can begin to piece together the dynamics of assembly processes over space and time, and under varying environmental conditions. These processes include both stochastic and deterministic mechanisms. Stochastic neutral theory assumes that species respond to chance colonization, extinction, and ecological drift, not because of their traits or that of their competitors, but rather because of random changes and thus have no interspecific trade‐offs (i.e., species are neutral) (Hubbell, 2001). Niche theory, on the other hand, assumes that site‐to‐site variations in species composition ares determined entirely by their specific traits, local habitat conditions, and interspecific relations, which in turn creates different niches that benefit different groups of species (determinism). The work of Chase and co‐workers, however, stipulates that the assembly of local communities is simultaneously driven by both deterministic and stochastic processes (Chase, 2010; Chase & Myers, 2011). Their framework is built around the concept that beta‐diversity patterns can resolve the relative influence of deterministic from stochastic processes in assembling the community structure along environmental gradients (e.g., space and time) (Chase & Myers, 2011).

**TABLE 1:** Brief summary of the null models used in this work

Null model	Description	Input	Reference
Normalized stochasticity ratio (NST)	A method that allows any type of beta‐diversity measure, whether it is incidence‐based (presence‐absence) or abundance‐based and gives a percentage of stochasticity for samples belonging to a single category	Any beta‐diversity metric as long as it is normalized to have a range between 0 and 1. Abundance‐based Ružička and incidence‐based Jaccard metrics were deemed to have a superior performance in view of the simulations done by the author	Ning et al. (2019)
Hill numbers	The effect of deterministic and stochastic factors on explaining the differences between multiple categories	Jaccard and Bray‐Curtis dissimilarity metrics	Modin et al. (2020)
β‐null deviation	Differentiation between niche and neutral community assemblage processes	Abundance‐based (Bray‐Curtis) β‐null deviation metric. Abundance‐based phylogenetic (UniFrac) measure	Tucker et al. (2016) Lee et al. (2017)
Quantitative Process Estimate (QPE)	Determination of the dominant assembly process between two given communities	Abundance table and phylogenetic tree on which βNTI and βRC are calculated for all pairs of samples	Stegen et al. (2013)
Lottery model	Determination of clade‐based community assembly where a single taxonomic group solely occupies a given niche due to a competitive advantage over other species (i.e., lottery winner)	Proportionally normalized abundance matrix	Verster and Borenstein (2018)
Phylogenetic dispersion	Characterization of the species distribution across treatments in relation to priority effects	Abundance table and phylogeny for samples that have temporal behavior which is captured as probability of recruitment. This is calculated as difference in phylogenetic diversity fitted with a logistic regression	Darcy et al. (2020)

Traditionally, large‐scale crude oil spills in the marine environment are treated with chemical dispersants to increase the surface‐to‐volume ratio of the oil, thereby increasing its biological availability to microorganisms and, consequently, enhancing its biodegradation (National Research Council of the National Academies, 2005). During the historic Deepwater Horizon oil spill that occurred in the Gulf of Mexico in 2010, approximately seven million liters of the dispersant Corexit were used in response to the release of an estimated 700,000 tonnes of Louisiana light crude oil (McNutt et al., 2012). Research into the response and diversity of marine hydrocarbon‐degrading microorganisms to this large spill revealed that dispersant exposure significantly altered microbial community dynamics and selected for specific taxa responding to hydrocarbons (Doyle et al., 2018; Dubinsky et al., 2013; Gutierrez et al., 2013; Miller et al., 2020; Redmond & Valentine, 2012). Furthermore, chemical dispersants were shown to inhibit oil degradation by suppressing some of the most effective hydrocarbon degraders such as *Marinobacter* and *Rhodococcus* (Hackbusch et al., 2020; Hamdan & Fulmer, 2011; Kleindienst et al., 2015; Rahsepar et al., 2016; Rughöft et al., 2020).

Biosurfactants, on the other hand, have for several decades been proposed as an effective, sustainable, and environmentally friendly alternative to chemical dispersants, but there remains a scarcity of available empirical data to substantiate this and, more specifically, their effect on microbial communities in the context of oil spills. In our previous work, we set up a microcosm experiment with chemically and biosurfactant‐dispersed crude oil in natural seawater from a subarctic region of the northeast Atlantic (Nikolova et al., 2020). Although having a similar community composition initially, the community dynamics in the biosurfactant‐dispersed oil microcosms became very distinct from that amended with chemically dispersed oil over time, suggesting that biosurfactants and dispersants select for different species. A similar outcome was observed in a more recent work comparing multiple chemical dispersants to rhamnolipid (Thomas et al., 2021). However, most studies investigating the effects of dispersant on microbial community have only relied on metagenomic and/or proteomic analyses to describe and measure the effect of dispersants on individual taxonomic groups (e.g., obligate hydrocarbon‐degrading species) and not on a community level in terms of assembly. The identification and quantification of ecological processes in microbial communities is relatively new concept with some of the model frameworks developed in the last 2 years.

In this study, we aimed to resolve patterns in the microbial community structure and assembly that improve our understanding of the role that chemical dispersants and biosurfactants play in the natural hydrocarbon biodegradation process. For this, we used 16S rRNA sequence data from our previous work and applied a set of recently developed null model frameworks (Table 1) which offer ecological insights.

## 2 MATERIALS AND METHODS

### 2.1 Sample collection, experimental set‐up, and barcoded 16S rRNA amplification

Field sample collection, microbial microcosms set‐up, and 16S rRNA amplicon Illumina sequencing used to perform the null models in this study have been described elsewhere in greater detail (Nikolova et al., 2020). Briefly, surface seawater was collected from the Faroe‐Shetland Channel (FSC) (60°16.36′N; 04°20.60′W). The seawater was used for the preparation of three main water accommodated fractions (WAFs) according to established protocols (Aurand & Coelho, 2005; Kleindienst et al., 2015). The first WAF contained seawater and crude oil only and is referred to as WAF. A Chemically Enhanced WAF (CEWAF) was prepared with seawater, crude oil, and addition of the synthetic dispersant Finasol OSR‐52 (Total Fluides, France) at a dispersant‐to‐oil ratio (DOR) of 1:20. Biosurfactant Enhanced WAF (BEWAF) was prepared with seawater, crude oil and rhamnolipid (at least 90% monorhamnolipids produced by *Pseudomonas aeruginosa*) at the same DOR as in the CEWAF. All three WAFs contained the same volume of filter‐sterilized seawater (1,560 ml) and Schiehallion crude oil (120 ml). In addition, two control WAFs were set up to contain only seawater and Finasol (SWD) or rhamnolipid (SWBS). After mixing for 48 hr, the three WAFs containing crude oil were allowed to stand undisturbed for 1 hr to allow for any undispersed oil to settle to the surface. The aqueous phase from each of the WAF mixtures was then carefully collected and used to prepare the microcosm treatments. Each treatment (in triplicates) contained 66 ml of the previously prepared WAF, CEWAF, BEWAF, SWD, or SWBS added to unfiltered seawater to a total volume of 300 ml in acetone‐rinsed, acid‐washed, and autoclaved 500‐ml glass bottles with Teflon‐lined caps, leaving 200 ml of head space to ensure aerobic conditions. All bottles were placed on a roller table to maintain constant gentle mixing (15 rpm) at 9.7℃ (in situ temperature at the time of sampling in the FSC) for 4 weeks in darkness to exclude the effect of photooxidation on the biodegradation of crude oil. At the beginning of these incubations (day 0), and then subsequently thereafter at days 3, 7, 14, and 28, each treatment was subsampled for DNA extraction following the method of Tillett and Neilan (2000).

### 2.2 High‐Throughput Sequencing and bioinformatics

DNA extraction, Illumina barcoded‐amplicon sequencing, and bioinformatic processing are described in detail elsewhere (Nikolova et al., 2020).

### 2.3 Microbial community assembly

Multiple null models (Table 1) were used for comprehensive and quantitative estimation of the fundamental ecological mechanisms driving community assembly. The null models were applied on the full amplicon sequences variants (ASVs) obtained after bioinformatic processing with the removal of reads belonging to mitochondria and chloroplast. The null models were performed in R v3.5.3, and Python and visualizations were conducted in R.

#### 2.3.1 Normalized stochasticity ratio (NST)

The NST for each treatment was calculated to measure the actual contribution of determinism in relation to stochasticity based on incidence‐based Jaccard and abundance‐based similarity Ružička metrics using null model algorithms of Taxa‐Richness constraints of proportional‐proportional (PP) and proportional‐fixed (PF), as recommended by Ning et al. (2019). For this, the “tNST” function from *NST* package in R was used (Ning et al., 2019). When using abundance‐based metric Ružička, null taxa abundances in each sample were calculated as random draw (1,000 times) of the observed number of individuals with probability proportional to regional relative abundances of null taxa in the treatments. The microbial community assembly is completely deterministic when NST is 0% and completely stochastic when NST is 100%. Permutational multivariate analysis of variance (PANOVA) was used to test whether the different treatments differed in their NST. Next, we calculated the NST of the seawater amended with oil (WAF), oil + dispersant (CEWAF), oil + biosurfactant (BEWAF), and of the nonamended seawater (SW) treatment over time to determine whether the proportion of stochasticity changed with time. The NST method becomes less precise when there are less than 6 replicates (Ning et al., 2019), and therefore, the temporal dynamics in each treatment were explored with Hill numbers null model.

#### 2.3.2 Hill numbers null model

Hill numbers are a set of diversity indices that can be plotted as continuous functions of the parameter q (Alberdi & Gilbert, 2019) representing the diversity order which determine the weight given to the relative abundance of ASVs in a community. Hill numbers null model framework used in this study was developed by Modin et al. (2020) and was implemented to quantify the dissimilarity between the microbial communities over time. For this, a Hill numbers beta‐diversity dissimilarity index ^q^ *d* was computed, with values scaled between 0 (similar) and 1 (dissimilar). The *^q^d* quantifies the effective average proportion of ASVs that are not shared between communities. Next, a null model generated a null version of a sample in which the number of ASVs was randomly picked from a real sample based on the frequency of samples in which the ASV was found and then populated the picked ASVs with reads equating to the total number of reads of the real sample. The randomization was applied 999 times to generate a null distribution for the dissimilarity between communities. If the values of observed dissimilarity (*^q^d*) and null distribution are similar, then stochastic factors are likely to cause the observed dissimilarity, and if higher or lower than the null distribution, deterministic factors that favor different or similar microbial taxa in two categories/treatments cause the observed dissimilarity. The calculation of *^q^d* and null distributions was performed in the qdiv package for Python3 (Modin et al., 2020).

#### 2.3.3 Tucker's beta‐null model

The beta‐null framework developed by Tucker et al. (2016) (hence the name Tucker's beta‐null model) was used to differentiate between neutral and niche communities. It is based on quantitative abundance‐based (Bray‐Curtis) dissimilarity matrix which was complemented by Lee et al. (2017) to integrate phylogenetic relatedness information with the taxonomic abundance (generalized UniFrac). The UniFrac dissimilarity metric was therefore used to calculate the beta‐null deviation values, which are indicative of the magnitude of deviation between the observed and expected beta‐diversity from randomly assembled pair of samples. The number of randomizations was set to 999. Beta‐null deviation values were calculated only for the three oil‐amended treatments (WAF, CEWAF, and BEWAF). The beta‐null deviation values between treatments for each time point were tested for significance with two‐way ANOVA and *post hoc* Tukey's test. Significance was accepted for *p*‐value <0.05.

#### 2.3.4 Quantitative process estimates (QPE)

QPE requires niche distances to correlate with phylogenetic distances (i.e., phylogenetic signal) so that phylogenetic turnover (the evolutionary distance differentiating taxa in one community from taxa in another community) can be estimated (Stegen et al., 2013). To perform QPE, the extent to which the abundance‐weighted ß‐mean‐nearest taxon distance (βMNTD) deviated from the mean of the null distribution was determined (after 999 randomizations) and the significance was evaluated by the ß‐Nearest Taxon Index (βNTI; difference between observed βMNTD and the mean of the null distribution in units of SDs). The presence of phylogenetic signal across short distances was assumed *post hoc* based on the observed significant values of βNTI as discussed elsewhere (Stegen et al., 2013; Trego et al., 2021). For instance, variable selection assembles the microbial community if the βNTI value is greater than 2. In contrast, the community is assembled by homogeneous selection when the βNTI is less than −2. In the case when there is no significant deviation from the null expectation, dispersal limitation, homogenizing dispersal (mass effect), or undominated processes (in which neither dispersal nor selection is the primary cause for compositional variations between communities) should drive the observed differences in phylogenetic community composition. To determine the relative importance of each of these processes, the abundance‐based (Raup‐Crick) beta‐diversity was calculated using pairwise Bray–Curtis dissimilarity metric (β_RCbray_) (Stegen et al., 2013).

#### 2.3.5 Lottery‐based assembly model

The lottery‐based assembly model was used to characterize the species distribution across treatments amended with crude oil with/without dispersant or rhamnolipid biosurfactant and to identify any microbial guilds whose distribution reflects a competitive lottery schema (Sale, 1979). The protocol developed by Verster and Borenstein (Verster & Borenstein, 2018) was followed with some exceptions. Briefly, the first step was to quantify how often species distribution within a group/guild includes a lottery winner (i.e., a group member that represent >90% of the group's abundance). The background expectation of the winner prevalence parameter was determined by implying a null model on the species abundances, which assumes a stick breaking process (Higgins & Strauss, 2008; MacArthur, 1957). Second, a measure of the diversity of lottery winners was calculated by the Shannon diversity of the distribution of winners across samples. This measure is referred to as the frequency at which each ASV occurs as the lottery winner among all samples/treatments in which lottery winner is observed. Diversity was normalized between 0 and 1 to account for differences in lottery winners. ASVs that had less than 5,000 reads appear at <0.05% abundance and had less than 3 ASVs were filtered out of the analysis.

#### 2.3.6 Phylogenetic dispersion model

The aim of the phylogenetic dispersion model was to estimate the extent of recruitment of new species in a microbial community over time based on how similar or dissimilar they are from previously recruited species (Darcy et al., 2020). The model characterized the probabilities of detecting new species in a local community over time and then simulated the data 500 times to produce surrogate datasets forward in time, which were then used to evaluate the null phylodispersity distribution *D*^ and the amount of phylodispersity (i.e., the sum of branch lengths on a phylogenetic tree for a set of species) accumulated over time PD_m_. A logistical error model (Darcy et al., 2020) is then performed to generate the dispersion parameter *D* which determined the extent to which either closely related or distant species were preferentially added to the surrogate community. *D* value >0 means that phylogenetically distant species are preferentially recruited in the local community (overdispersion; phylogenetically divergent), whereas *D* < 0 indicate the opposite—phylogenetically similar species are detected in the local community (underdispersion; phylogenetically constrained). If *D* = 0, all species have the same probability of being detected for the first time (neutral).

## 3 RESULTS

### 3.1 Stochastic versus deterministic assembly processes on a temporal scale

**FIGURE 1:**
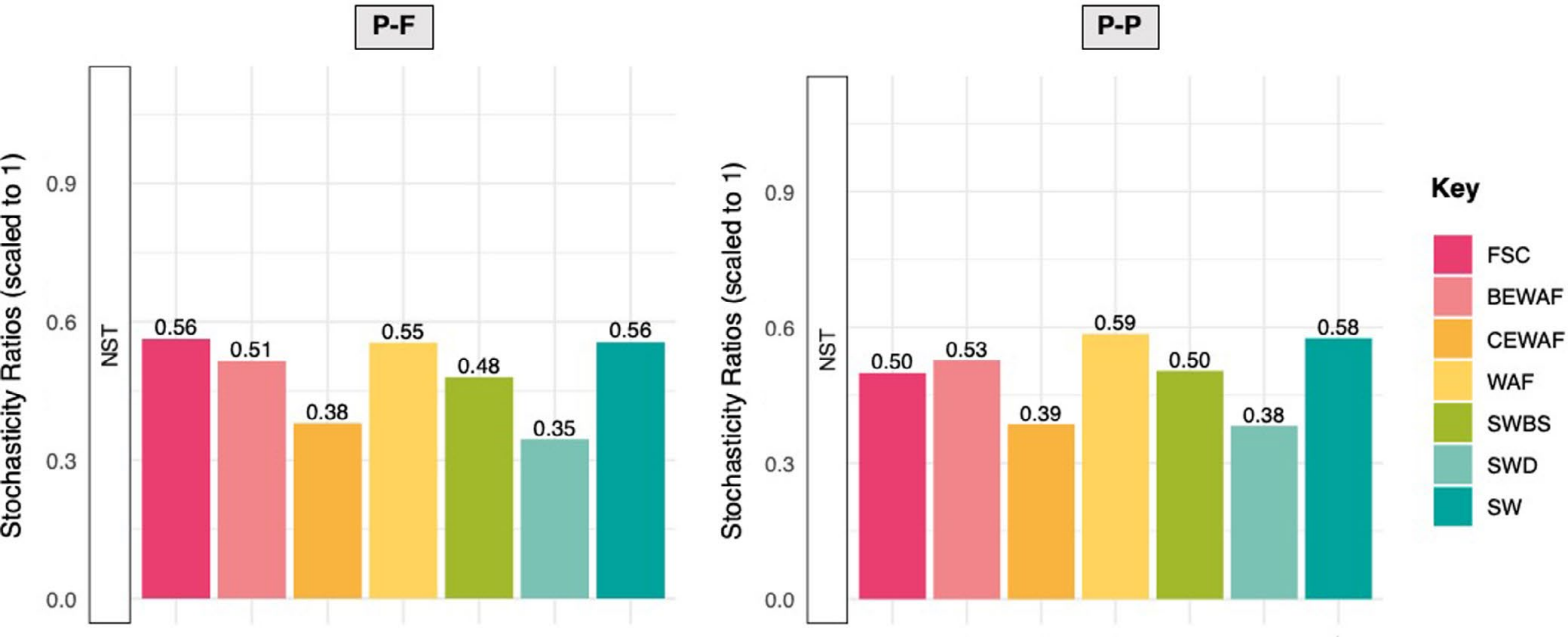
Normalized stochasticity ratio (NST) calculated based on abundance‐based Ružička metric and Taxa‐Richness constraints of proportional‐fixed (P‐F) and proportional‐proportional (P‐P) which stipulates that the probabilities of taxa occurrence are proportional to the observed occurrence frequencies, and taxon richness in each sample is either fixed or proportional. Treatments: in situ seawater (FSC), crude oil‐amended seawater (WAF), crude oil +dispersant‐amended seawater (CEWAF), crude oil + rhamnolipid‐amended seawater (BEWAF), seawater amended with Finasol (SWD) or rhamnolipid (SWBS), and nontreated seawater (SW; control)

In general, the NST revealed that the microbial community assembly in the studied treatments were neither purely deterministic nor purely stochastic as indicated in Figure 1. The in situ FSC community, the crude oil and seawater WAF treatment, and the seawater only control (SW) had the same NST value of 56%, while the rhamnolipid‐amended oiled seawater treatment (BEWAF) had a NST of 51%, suggesting that the communities' assembly in these treatments was driven slightly more by stochastic processes. Communities in the treatments with added chemical dispersant Finasol (CEWAF and SWD), in contrast, were dominated by deterministic processes as their NST was, respectively, 38% and 35%. PANOVA analysis revealed that the CEWAF treatment was significantly different from the SW control treatment (*p* = .019) and the SWD treatments was significantly different from the BEWAF (*p* = .038), WAF (*p* = .018), and the SW (*p* = .002) treatments. The rest of the treatments were not found to be significantly different from each other (Table S1).

**FIGURE 2:**
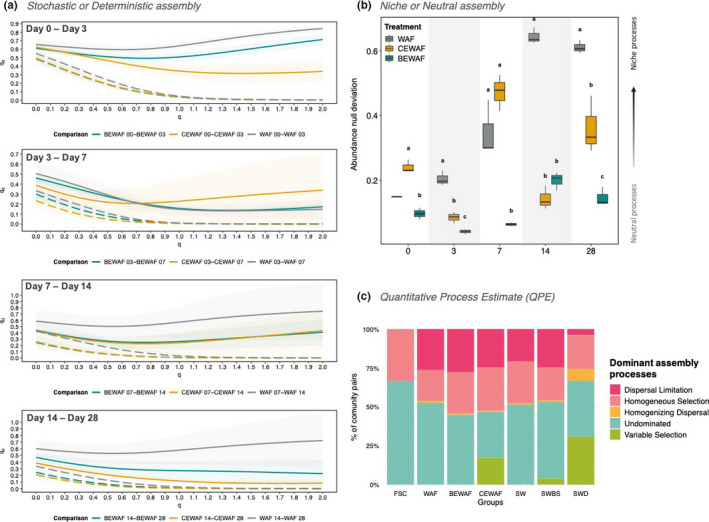
Presentation of four themes of community assembly processes (Stochasticity, Determinisms, Niche, and Neutrality) and quantitative measures of specific ecological processes. (a) Hill‐based dissimilarity (solid lines) and the null expectation (dashed lines) based on 999 randomizations for treatment groups (BEWAF, CEWAF, and WAF) pairwise comparisons: day 0 versus day 3, day 3 versus day 7, day 7 versus day 14, and day 14 versus day 28. Shaded regions show standard deviation based on all pairwise comparisons (*n* = 45). (b) Relative changes in niche and neutral processes assessed with abundance‐weighted phylogenetic (generalized UniFrac) deviation from beta‐null model for treatments WAF, CEWAF, and BEWAF over time (days 0–28). Significance between group means for each time point was tested with Two‐way ANOVA analysis and *post hoc* Tukey's test. Groups that share different letters are significantly different from each other. (c) Relative importance of species‐sorting (variable or homogeneous selection), dispersal limitation or historical contingency, homogenizing dispersal or undominated community assembly processes for all treatment groups

Next, we looked at the temporal dissimilarity between the microbial communities in BEWAF, CEWAF, and WAF treatments for a range of diversity orders using Hill‐based indices and compared them to the null expectation (Figure 2a). The observed temporal dissimilarity between day 0 and day 3 had *^q^d* values >0.8, 0.7, and 0.3 for WAF, BEWAF, and CEWAF, respectively. However, as time progressed, the BEWAF and CEWAF communities showed less dissimilarity from the null distribution than the WAF community, and therefore, the null expectation of random (i.e., stochastic) community assembly in BEWAF and CEWAF could not be rejected. During the first seven days of incubation, the microbial communities in the BEWAF and WAF treatments were more similar to each other than either was to the CEWAF. However, during the second half of the incubation period (days 7–14, and days 14–28), the CEAWF and BEWAF communities were more similar to each and more dissimilar to the WAF community (Figure 2a). Collectively, the NST and Hill numbers approaches showed that stochastic processes could play more important roles in controlling community succession in its mid‐phase and late phase, while deterministic processes could be more important during the early phase. A similar trend was also observed for the control treatments SWBS, SWD, and SW (Figure S1).

### 3.2 Tucker's beta‐null model

Next, we quantified the relative contribution of niche and neutral processes in the assembly of the bacterial communities in seawater microcosms amended with crude oil (WAF) and dispersant (CEWAF) or rhamnolipid (BEWAF). The WAF treatment deviated from the null expectation toward a significant increase in the contribution of niche processes in the community assembly by the end of the incubation time period (Figure 2b). In contrast, the relative contribution of niche processes in the BEWAF and CEWAF microcosms was more variable over time. The analysis of the weighted UniFrac deviation from null model indicated that the relative contribution of neutral processes was more dominant in the BEWAF treatments assembly over the late phase of the studied period (days 14–28) (Figure 2b). BEWAF's Bray–Curtis null deviation was significantly different from that of the CEWAF on day 28, indicating that phylogenetically distinct ASVs between the two treatments might play a role in shaping the bacterial community assembly toward more niche or more neutral processes.

### 3.3 Quantitative process estimates (QPE)

The aim of the QPE was to estimate the relative importance of selection driven by deterministic processes, dispersal driven by stochastic processes resulting in drift, and undominated processes. Considering the overall dynamics of these processes in the different treatments, there are some clear differences. For the in situ FSC community, random undominated processes were the dominant assembly process (67%) followed by homogeneous selection (33%) (Figure 2c). On the other hand, in the rest of the treatments, the dominant assembly processes were undominated processes (ranging 29%–52%), homogeneous selection (19%–27%), and dispersal limitation (3%–27%). Undominated processes were the least important in the CEWAF (29.5%) and the SWD (35.9%) treatments, while in the WAF and seawater control (SW) treatments they dominated the assembly in more than 50% of community pairs (Figure 2c). Homogeneous selection had similar relative importance in all of the treatments (except the in situ FSC). Dispersal limitation was similarly important in the assembly of communities in all treatments (20.9%–27.6%) except for the in situ FSC community and the SWD treatment where the dispersal limitation was not important at all (0%) or in very small proportion (3.8%), respectively. Among all treatments, variable selection was relatively important in only two treatments—CEWAF (17%) and SWD (30.7%). Next, we plotted the temporal profiles for QPE for each treatment, although limited to only those treatments which have more than two replicates available (Figure S2). Generally, undominated processes were the most dominating throughout the incubation period for all treatments. However, it is important to note that, other than cross comparing the ßNTI estimates for multiple categories indirectly, the QPE method is not designed to incorporate temporal behavior directly, and, hence, the temporal assembly variation cannot be reliably explained with this method.

### 3.4 Competitive lottery‐controlled genera

**FIGURE 3:**
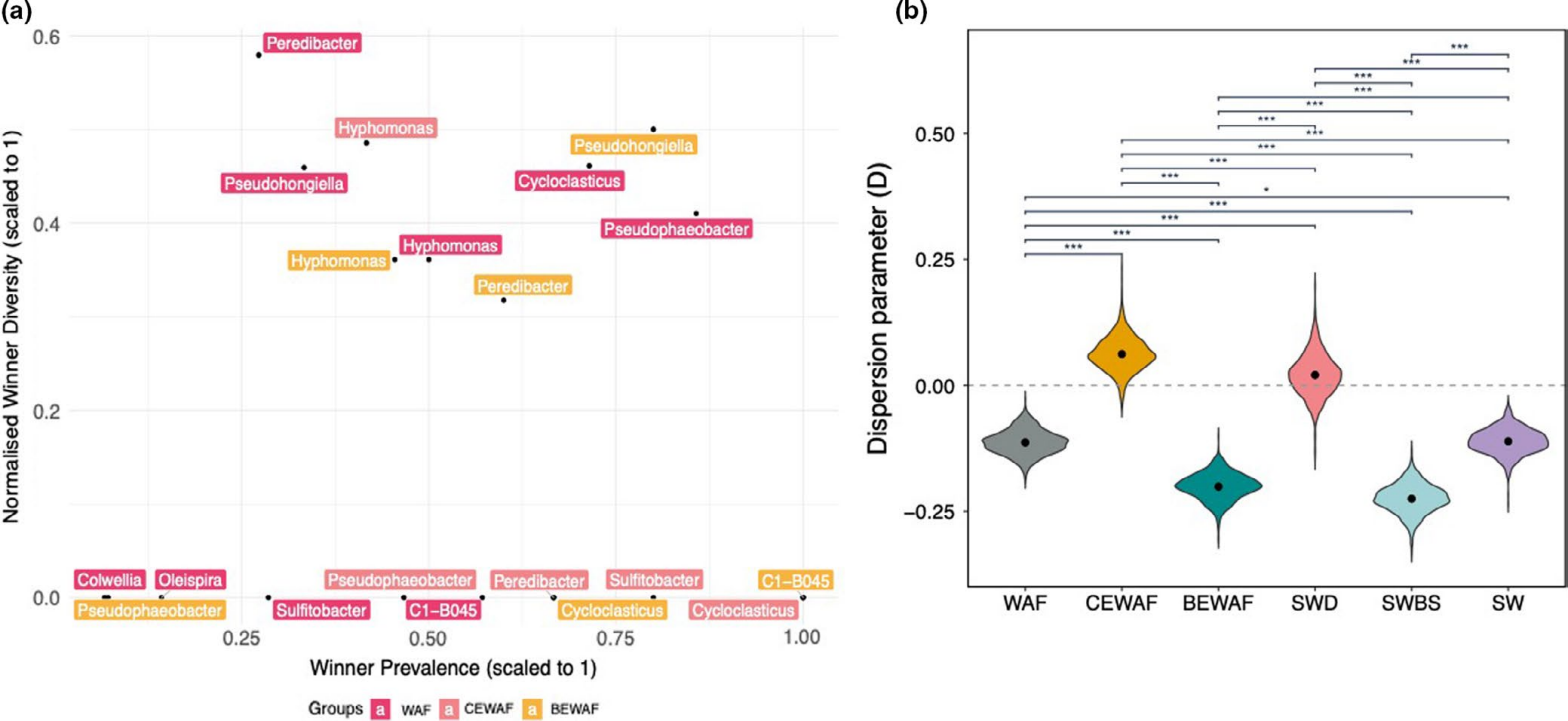
(a) A scatter plot showing the winner prevalence and winner diversity for different genera in three crude oil‐amended seawater treatments: WAF (oil + seawater), CEWAF (oil + seawater + Finasol), and BEWAF (oil + seawater + rhamnolipid). (b) Distribution of dispersion parameter (*D*) estimates given by logistic error model bootstrap for six treatments (fill color). Dots within each violin are means. Treatments are seawater (SW) amended with crude oil (WAF), crude oil and dispersant Finasol (CEWAF), crude oil and rhamnolipid biosurfactant (BEWAF), Finasol only (SWD), or rhamnolipid only (SWBS) over time. Significantly different (*t* test) treatments share a bracket, and the level of significance is shown with * (*p* = .05), ** (*p* = .01), or *** (*p* = .001)

**FIGURE 4:**
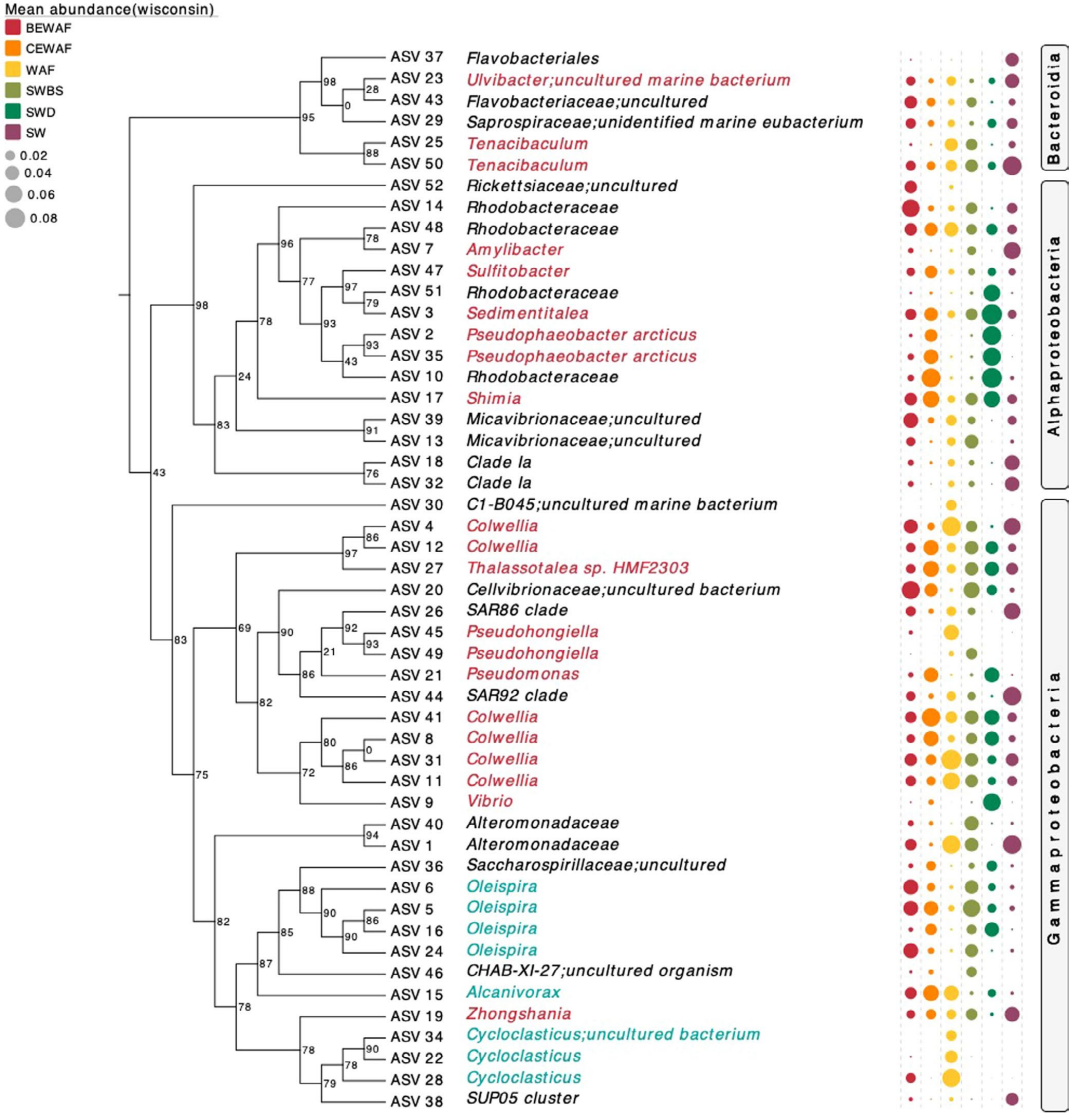
Phylogenetic tree showing the top 50 most abundant ASVs which have been taxonomically assigned with SILVA SSU v132 database. ASVs in red font represent taxa with known generalist hydrocarbonoclastic bacteria, and ASVs in blue font represent obligate hydrocarbonoclastic bacteria. Dot plots on the right side of the tree show the mean abundance of each ASV colored by treatment after performing proportional standardization using the Wisconsin function. Numbers in nodes represent bootstrap values. The tree was visualized with the web tool Evolview2

The distribution of species across treatments and identity of lottery “winners” were characterized with the help of the lottery‐based assembly model. The ASV distribution was expected to display two fundamental features. First, a single group member captures >90% of the group's abundance (the “lottery winner”) in each treatment, and, second, different treatments should have different lottery winners. The winner prevalence (the fraction of samples in which one ASV was assigned >90% of the genus abundance) and the winner diversity for each genus in the WAF, CEWAF, and BEWAF treatments were plotted in Figure 3a. *Pseudophaeobacter* was the lottery winner in the WAF treatment as it was present in 85% of samples and showed highest winner diversity (41%). Similarly, one ASV of *Cycloclasticus* was in 71% of the WAF samples but it had a higher winner diversity than *Pseudophaeobacter*. The lottery winner in the BEWAF treatment was *Pseudohongiella* which was present in 80% of the samples. In the CEWAF treatment, the genus with the highest winner prevalence was a different *Cycloclasticus* ASV (from the one in the WAF treatment) but it had a winner diversity of 1, meaning that this particular *Cycloclasticus* ASV was not consistent with the competitive lottery schema. Groups that have low winner prevalence and comparatively higher winner diversity likely reflected that the group abundances were more evenly distributed among the group ASVs (e.g., *Peredibacter*, *Hyphomonas*). Interestingly, however, all lottery winners had very low relative abundance in their respective microcosms (Figure 4).

### 3.5 Phylogenetic dispersion

The phylogenetic dispersion model provided a view into temporal dynamics in the recruitment of new species in the local community in relation to their phylogenetic similarity or dissimilarity to already existing members of the community which colonized it at a previous time point (Darcy et al., 2020). Varying *D* ^^^ changed the rate at which the phylodiversity was added to the resampled microbial communities over time (Figure 3b), and overall, the results showed that the *D* parameter successfully corresponded to over‐ and underdispersion relative to the neutral model (Figure S3). The CEWAF treatment had the highest *D* value (*D* > 0) compared to the other two oil‐amended treatments (WAF and BEWAF), indicating that the presence of dispersant changed the phylogenetic colonization patterns to a preferential and faster recruitment of phylogenetically distant species in the community (i.e., overdispersion). In contrast, the WAF and BEWAF treatments had *D* value of less than 0 which suggests that there was underdispersion or phylogenetically similar new species were detected in the local community. In other words, the addition of dispersant caused the local community to become phylogenetically divergent as time progressed, whereas the addition of rhamnolipid caused the opposite trend —that is, a phylogenetically constrained community. The oil by itself (WAF) also caused a constrained community but less so compared to the rhamnolipid‐amended oil treatment (BEWAF). All treatments had significantly different *D* values (*p* < .001) (Figure 3b).

## 4 DISCUSSION

### 4.1 Stochastic versus deterministic assembly

It has been generally accepted that both deterministic and stochastic processes occur simultaneously in the assembly of local communities (Chase et al., 2011). Bacterial communities across all communities studied here were neither purely stochastic nor purely deterministic. In particular, the NST showed that the oil‐only control treatment (WAF) and untreated seawater control (SW) had more stochastically assembled communities, while the communities in the treatments with added dispersant Finasol (CEWAF and SWD) were more deterministic. It is likely that the presence of dispersant triggered a microbial response related to deterministic succession. Furthermore, Hill numbers model revealed that the relative importance of stochasticity over determinism varied substantially over time. Stochastic processes were more prevalent in the mid‐phase and late phase of incubation, while deterministic processes are more important in the early phase (within 3 days) in all treatments. This result seems to fit the intuition that adding carbon source (e.g., crude oil and/or dispersant/biosurfactant) or altering the environmental conditions should drive selection and hence leads to a more deterministic outcome in the beginning, and as disturbance effect is weakened (e.g., the oil is increasingly biodegraded), stochasticity would increase near to the original level (Zhou et al., 2014). This is a demonstration that drivers controlling biodiversity and community succession are dynamic rather than static in oil‐polluted fluidic ecosystems (Dini‐Andreote et al., 2015; Zhou et al., 2014).

### 4.2 Neutral versus niche assembly

The Tucker's beta‐null deviation model successfully differentiated patterns of niche and neutral processes in the three crude oil‐amended treatments (WAF, CEWAF, and BEWAF) in the presence of either dispersant or rhamnolipid over time, suggesting that the presence of dispersant and rhamnolipid did have an important role or even were selective factors in the community assembly processes. Neutral processes had a more prominent role in the assembly in the rhamnolipid‐amended oil treatment (BEWAF) than in the oil‐only and Finasol‐amended oil treatment (CEWAF). This finding suggests that the addition of rhamnolipid had not applied strong selection on the assembly of oiled seawater microcosms. The opposite, and contrary to the NST results, was observed for the oil‐only treatment (WAF)—clear niche community assembly pattern. A plausible explanation is that the oil selected for highly specialized species, while the dispersant and rhamnolipid allowed more generalist taxa to thrive (Nikolova et al., 2020). The difference between the observation drawn from the NST and Tucker's beta‐null model can be attributed to the different dissimilarity metrics used to calculate the null deviation values. While the NST approach is built upon abundance‐based matrix, the Tucker's null model was modified to integrate phylogenetic relatedness information with the taxonomic abundance and, hence, increase our confidence in the cause of the observed dissimilarity between treatments. One disadvantage of the beta‐null model is that it does not reveal exactly what processes affect the community.

### 4.3 Importance of selection and dispersal

According to QPE, all treatments were dominated by dispersal limitation, homogeneous selection, and undominated mechanisms (weak selection and moderate dispersal). Generally, selection has more detectable influence over microbial communities (Ning et al., 2019; Stegen et al., 2013; Zhou & Ning, 2017) and the same was observed in this study. The importance of variable selection was substantially higher only in the treatment containing Finasol dispersant (CEWAF and SWD). In fact, the variable selection causes an increase in the spatial environmental heterogeneity as time (i.e., succession) progresses which leads to compositional differences across local communities (Dini‐Andreote et al., 2015). As expected, homogenizing dispersal was not an important factor in the community assembly in our closed‐system microcosms. However, in open marine ecosystems where there is a rapid dispersion and population movement (i.e., high connectivity) (Langenheder & Ragnarsson, 2007; Zhou et al., 2014), dispersal is expected to play a stronger role. Undominated processes were the most dominant in the majority of treatments but notably lower in both Finasol‐amended treatments (CEWAF and SWD). The dispersal limitation was relatively higher than it would be expected for open ocean ecosystems, likely because the microbial microcosms in this study were enclosed in bottles and thus spatially differentiated from each other with no direct dispersal occurring. For this reason, it is important to be mindful that interpretation of the relative importance of dispersal limitation in the context of this study should be done with caution. There is growing evidence, however, to support that the dispersal limitation can actually be a more important dominating factor in marine microbial communities than previously thought, especially when acting together with drift (Dumbrell et al., 2010; Stegen et al., 2015b). Other microbial studies, including in close systems such as anaerobic digestors (Trego et al., 2021) that used quantitative process estimates, have demonstrated the substantial proportion of the dispersal limitation or historical contingency (such as priority effects) in community assembly (Langenheder et al., 2017; Vass et al., 2020; Wu et al., 2018). It is reasonable to suggest that in an oil spill in the field, dispersant or biosurfactant application would not have a strong deterministic effect due to the higher dispersal rates observed in oceanic systems. Stochasticity is influenced by an interaction between dispersal and selection, with stronger selection causing an increase in drift or priority effects (Evans et al., 2017). The importance of drift is considered higher when selection is weak, and the local community size is small (Vellend, 2010). Until recently, pure drift was practically impossible to measure, and even more so for microbial communities where the other ecological processes act simultaneously (Vellend, 2010). However, in controlled experiments on simplified synthetic bacterial communities, drift was successfully quantified via direct observations at the population level using an experimental set‐up that isolates drift from other processes and subtracts the experimental and methodological noise (Fodelianakis et al., 2020).

### 4.4 Lottery winners

The lottery winner ASVs varied between treatments in accordance with the competitive lottery schema. Furthermore, the lottery winners in each treatment did not occur at the same frequency as assumed by the lottery schema (Verster & Borenstein, 2018). A winner diversity approaching 0 means that the same species is selected in all cohorts for a given clade. There were a number of genera with very high winner prevalence but very low winner diversity, including *Cycloclasticus* and *Sulfitobacter*, that did not involve complete competition‐derived exclusion but rather strong coexistence with other species in the microcosms or even more complex assembly that combines exclusion and coexisting patterns (Verster & Borenstein, 2018). Taking into consideration the relative abundance of the top 25 most abundant ASVs observed in Nikolova et al. (2020), it became apparent that the lottery winners in each treatment were not necessarily the most abundant species identified. For example, *Cycloclasticus* was identified as a potential lottery winner in the WAF treatment. *Cycloclasticus*, which is a polycyclic aromatic hydrocarbon (PAH) degrader, was found to dominate the WAF's community composition in the late stages of incubation, while early stages were dominated by *Colwellia* and *Oleispira*, which are aliphatic (e.g., alkanes) and low‐molecular‐weight PAH degraders. It is logical then to assume that microbial succession, driven by competition for resources, occurred in the WAF treatment—that is, *Cycloclasticus* outcompeted *Colwellia* and *Oleispira*. This succession of aliphatic hydrocarbon degraders becoming enriched initially and then being followed by PAH degraders is a common occurrence during oil spills at sea (Head et al., 2006). Furthermore, *Cycloclasticus* had a relative abundance of <1% across all CEWAF samples and it is highly likely that just one ASV was entirely responsible for the observed low abundance. According to the lottery‐based assembly model, a single *Cycloclasticus* ASV was present in 100% of all samples in the CEWAF treatments but did not fit the assumptions of the competitive lottery schema. This suggests that this particular *Cycloclasticus* ASV was either outcompeted by other species (potentially more adept to the presence of dispersant or able to engage in resource partitioning) observed in the CEWAF treatment (e.g., by *Rhodobacteraceae*) to the point where its abundance never exceeded 1%, or the presence of the dispersant Finasol itself might have selected against it. Interestingly, generalist species *Pseudophaeobacter* and *Pseudohongiella* were projected the lottery winners in the WAF and BEWAF treatments, respectively, but both had less than 1% abundance in the respective treatments (Nikolova et al., 2020). In fact, *Pseudophaeobacter* represented up to 40‐fold higher abundance in the CEWAF treatment, while *Pseudohongiella* was most abundant in the WAF treatment. It is possible that there was other more complex assembly schema (which the lottery‐based assembly method could not explain) that *Pseudophaeobacter* and *Pseudohongiella* conformed to in comparison to *Cycloclasticus*. For example, although they were lottery winners, both species could have facilitated subsequent species that “join” the ecosystem (i.e., respond to crude oil) to flourish. It is also possible that as generalists, *Pseudophaeobacter* and *Pseudohongiella* have more opportunities for niche diversification, outcompeting the rest of the community members.

## 5 PHYLOGENETIC DISPERSION

The phylogenetic dispersion model is the most recently developed null model and hence, it has not been tested in other environmental microbial studies. Nevertheless, the model provides a valuable insight into the largely unknown area of how and when microbial communities are colonized by phylogenetically similar or dissimilar relatives (Darcy et al., 2020). Microcosm treatments with added dispersant Finasol (CEWAF and control SWD) were the only two treatments that had *D* values >0 indicating that their microbial community had become more phylogenetically divergent as time progressed and there was little preference of which species were recruited first (i.e., random colonization). The rest of the treatments (WAF, BEWAF, and the controls SWBS and SW) followed a “nepotistic” pattern of new species recruitment. This “nepotistic” pattern was described as a recruitment pattern in which new species that are closely related to the already existing member of the community are more likely to be recruited than distantly related species (D’Andrea et al., 2019; Darcy et al., 2020). Traditionally, community ecology assumes that competition among closely related species would be strongest but also allows similar species to coexist, especially when dispersion is high as is in ocean ecosystems (D’Andrea et al., 2019). One explanation for the observed overdispersion in the CEWAF treatment is that the dispersant caused the formation of multiple environmental gradients in the community which provided an opportunity for different, and arguably more functionally diverse, species to colonize the community rather than explicitly promoting functionally similar taxa such as obligate hydrocarbon degraders. The multiple environmental gradients can be attributed to the added chemical complexity of the dispersed crude oil cause by the addition of the dispersant. Furthermore, the QPE model showed that variable selection was only observed in the microcosms with added chemical dispersant. Variable selection in the microcosms with added biosurfactant and oil‐only was not detected and, hence, explains the observed phylogenetic underdispersion which posits that recruitment of new species is slow, that is, there is a single environmental gradient available to colonize. This is somewhat opposite to the Tucker's beta‐null model which showed that the community in the BEWAF treatment was assembled more by neutral processes than the CEWAF treatment.

In summary, the combination of null models revealed marked differences in microbial consortia structure between the dispersant‐ and the biosurfactant‐amended microcosms and have unveiled insightful structures indicative of the underlying ecological processes. Specifically, according to the NST and QPE, neither purely deterministic nor purely stochastic processes drive the community assembly, but determinism was slightly stronger in the microcosms with chemical dispersant. The Hill numbers approach highlighted that the influence of stochasticity and determinism was dynamic over time in all microcosms, confirming that in aquatic systems stochastic processes have more weight in the community assembly following the addition of nutrients, while determinism drive the assembly in the early stages. In contrast, the beta‐null approach revealed that niche and neutrality processes were highly dynamic but overall, the WAF community was more divergent (niche), and the CEWAF and BEWAF had communities in which species are likely to be ecologically equivalent. This is in a disagreement with the phylogenetic dispersion approach which showed that the CEWAF community is phylogenetically divergent while the BEWAF and WAF treatments likely supported closely related species. The taxonomic composition analysis (see Nikolova et al., 2020), however, revealed that Finasol played a major role in influencing the microbial dynamics by negatively impacted diversity and selection for generalist and opportunistic species (e.g., *Rhodobacteracaea* and *Vibrionaceae*) with flexible or variable functional response that can modulate the community functional profile in a desired direction (e.g., depending on environmental conditions, oil biodegradation can be either enhanced or inhibited). This effect was stronger than what was observed in oil‐only (WAF) and rhamnolipid‐amended oil treatments (BEWAF), which supported higher diversity of obligate hydrocarbon‐degrading taxa (e.g., *Oleispira*, *Alcanivorax*, and *Cycloclasticus*) and potentially being more efficient in biodegrading hydrocarbons over time. Based on this observation, we hypothesize that the domination of generalist/opportunistic taxa caused by the chemical dispersant in the community could be the major driver of determinism. In addition, the most abundant species were not the “lottery winners” in the communities but rather those that are resilient to environmental changes (i.e., introduction of oil and dispersant or biosurfactant). In view of our findings, we recommended to apply various null models and then find the consensus agreement or consistent patterns among them. This is particularly important as the methods capture different aspects of microbial consortia, whether focusing on abundances, or on phylogeny, and may also have analytical biases. Although this study was performed on laboratory‐scaled microcosms, the measured mechanisms revealed assembly patterns that are generally consistent with theoretical assumptions and empirical data from other microbial studies. Nevertheless, the microbial ecology of full‐scale marine systems contaminated with crude oil (i.e., offshore oil spill) is more complex with respect to environmental conditions, oceanographic dynamics, and nutrient availability, and therefore, microbial communities in such systems are expected to be more dominated by dispersal processes than under controlled laboratory conditions.

## CONFLICT OF INTEREST

None declared.

## AUTHOR CONTRIBUTIONS

**Christina Nikolova:** Conceptualization (supporting); data curation (lead); formal analysis (equal); project administration (equal); visualization (equal); writing–original draft (lead); writing–review and editing (equal). **Umer Zeeshan Ijaz:** Conceptualization (lead); funding acquisition (equal); methodology (equal); resources (lead); software (lead); supervision (equal); writing–review and editing (equal). **Tony Gutierrez:** Funding acquisition (equal); project administration (equal); supervision (equal); writing–review and editing (equal).

## Supporting information

Supplementary Material

Appendix S1

Appendix S2

## Data Availability

The raw 16S rRNA sequences are available in the NCBI SRA database under accession number PRJNA636672. DOI: https://doi.org/10.5061/dryad.s7h44j17b.

## References

[ece38091-bib-0001] Alberdi, A. , & Gilbert, M. T. P. (2019). A guide to the application of Hill numbers to DNA‐based diversity analyses. Molecular Ecology Resources, 19, 804–817. 10.1111/1755-0998.13014 30947383

[ece38091-bib-0002] Amann, R. I. , Ludwig, W. , & Schleifer, K. H. (1995). Phylogenetic identification and in situ detection of individual microbial cells without cultivation. Microbiological Reviews, 59, 143–169. 10.1128/mr.59.1.143-169.1995 7535888 PMC239358

[ece38091-bib-0003] Aurand, D. , & Coelho, G. M. (2005). Cooperative aquatic toxicity testing of dispersed oil and the chemical response to oil spills: Ecological Effects Research Forum (CROSERF). Inc. Lusby, MD. Tech. Report, 07‐03.

[ece38091-bib-0004] Azam, F. , Fenchel, T. , Field, J. G. , Gray, J. S. , Meyer‐Reil, L. A. , & Thingstad, F. (1983). The ecological role of water‐column microbes in the sea. Marine ecology progress series, 257–263.

[ece38091-bib-0005] Chase, J. M. (2010). Stochastic community assembly causes higher biodiversity in more productive environments. Science, 328, 1388–1391. 10.1126/science.1187820 20508088

[ece38091-bib-0006] Chase, J. M. , Kraft, N. J. B. , Smith, K. G. , Vellend, M. , & Inouye, B. D. (2011). Using null models to disentangle variation in community dissimilarity from variation in α‐diversity. Ecosphere, 2. 10.1890/ES10-00117.1

[ece38091-bib-0007] Chase, J. M. , & Myers, J. A. (2011). Disentangling the importance of ecological niches from stochastic processes across scales. Philosophical Transactions of the Royal Society B: Biological Sciences, 366, 2351–2363. 10.1098/rstb.2011.0063 PMC3130433 21768151

[ece38091-bib-0008] D’Andrea, R. , Riolo, M. , & Ostling, A. M. (2019). Generalizing clusters of similar species as a signature of coexistence under competition. PLoS Computational Biology, 15, 1–19. 10.1371/journal.pcbi.1006688 PMC6358094 30668562

[ece38091-bib-0009] Darcy, J. L. , Washburne, A. D. , Robeson, M. S. , Prest, T. , Schmidt, S. K. , & Lozupone, C. A. (2020). A phylogenetic model for the recruitment of species into microbial communities and application to studies of the human microbiome. ISME Journal, 14, 1359–1368. 10.1038/s41396-020-0613-7 PMC7242462 32076128

[ece38091-bib-0010] Dini‐Andreote, F. , Stegen, J. C. , Van Elsas, J. D. , & Salles, J. F. (2015). Disentangling mechanisms that mediate the balance between stochastic and deterministic processes in microbial succession. Proceedings of the National Academy of Sciences of the United States of America, 112, E1326–E1332. 10.1073/pnas.1414261112 25733885 PMC4371938

[ece38091-bib-0011] Doyle, S. M. , Whitaker, E. A. , De Pascuale, V. , Wade, T. L. , Knap, A. H. , Santschi, P. H. , Quigg, A. , & Sylvan, J. B. (2018). Rapid formation of microbe‐oil aggregates and changes in community composition in coastal surface water following exposure to oil and the dispersant Corexit. Frontiers in Microbiology, 9, 689. 10.3389/fmicb.2018.00689 29696005 PMC5904270

[ece38091-bib-0012] Dubinsky, E. A. , Conrad, M. E. , Chakraborty, R. , Bill, M. , Borglin, S. E. , Hollibaugh, J. T. , Mason, O. U. , M. Piceno, Y. , Reid, F. C. , Stringfellow, W. T. , & Tom, L. M. (2013). Succession of hydrocarbon‐degrading bacteria in the aftermath of the deepwater horizon oil spill in the Gulf of Mexico. Environmental Science and Technology, 47, 10860–10867. 23937111 10.1021/es401676y

[ece38091-bib-0013] Dumbrell, A. J. , Nelson, M. , Helgason, T. , Dytham, C. , & Fitter, A. H. (2010). Relative roles of niche and neutral processes in structuring a soil microbial community. ISME Journal, 4, 337–345. 10.1038/ismej.2009.122 19924158

[ece38091-bib-0014] Evans, S. , Martiny, J. B. H. , & Allison, S. D. (2017). Effects of dispersal and selection on stochastic assembly in microbial communities. ISME Journal, 11, 176–185. 10.1038/ismej.2016.96 PMC5315486 27494293

[ece38091-bib-0015] Fodelianakis, S. , Valenzuela‐Cuevas, A. , Barozzi, A. , & Daffonchio, D. (2020). Direct quantification of ecological drift at the population level in synthetic bacterial communities. ISME Journal, 15, 55–66. 10.1038/s41396-020-00754-4 PMC7852547 32855435

[ece38091-bib-0016] Gutierrez, T. , Singleton, D. R. , Berry, D. , Yang, T. , Aitken, M. D. , & Teske, A. (2013). Hydrocarbon‐degrading bacteria enriched by the Deepwater Horizon oil spill identified by cultivation and DNA‐SIP. ISME Journal, 7, 2091–2104. 10.1038/ismej.2013.98 PMC3806270 23788333

[ece38091-bib-0017] Hackbusch, S. , Noirungsee, N. , Viamonte, J. , Sun, X. , Bubenheim, P. , Kostka, J. E. , Müller, R. , & Liese, A. (2020). Influence of pressure and dispersant on oil biodegradation by a newly isolated Rhodococcus strain from deep‐sea sediments of the Gulf of Mexico. Marine Pollution Bulletin, 150. 10.1016/j.marpolbul.2019.110683 31753565

[ece38091-bib-0018] Hamdan, L. J. , & Fulmer, P. A. (2011). Effects of COREXIT EC9500A on bacteria from a beach oiled by the Deepwater Horizon spill. Aquatic Microbial Ecology, 63, 101–109. 10.3354/ame01482

[ece38091-bib-0019] Hassani, M. A. , Özkurt, E. , Franzenburg, S. , & Stukenbrock, E. H. (2020). Ecological assembly processes of the bacterial and fungal microbiota of wild and domesticated wheat species. Phytobiomes Journal, 4(3), 217–224. 10.1094/PBIOMES-01-20-0001-SC

[ece38091-bib-0020] Head, I. M. , Jones, D. M. , & Röling, W. F. (2006). Marine microorganisms make a meal of oil. Nature Reviews Microbiology, 4(3), 173–182. 16489346 10.1038/nrmicro1348

[ece38091-bib-0021] Higgins, C. L. , & Strauss, R. E. (2008). Modeling stream fish assemblages with niche apportionment models: Patterns, processes, and scale dependence. Transactions of the American Fisheries Society, 137, 696–706. 10.1577/T07-061.1

[ece38091-bib-0022] Hubbell, S. P. (2001). The unifed neutral theory of biodiversity and biogeograophy (MPB‐32). Princeton University Press.

[ece38091-bib-0023] Kleindienst, S. , Seidel, M. , Ziervogel, K. , Grim, S. , Loftis, K. , Harrison, S. , Malkin, S. Y. , Perkins, M. J. , Field, J. , Sogin, M. L. , Dittmar, T. , Passow, U. , Medeiros, P. M. , & Joye, S. B. (2015). Chemical dispersants can suppress the activity of natural oil‐degrading microorganisms. Proceedings of the National Academy of Sciences, 112, 14900–14905. 10.1073/pnas.1507380112 PMC4672791 26553985

[ece38091-bib-0024] Langenheder, S. , & Ragnarsson, H. (2007). The role of environmental and spatial factors for the composition of aquatic bacterial communities. Ecology, 88, 2154–2161. 10.1890/06-2098.1 17918394

[ece38091-bib-0025] Langenheder, S. , Wang, J. , Karjalainen, S. M. , Laamanen, T. M. , Tolonen, K. T. , Vilmi, A. , & Heino, J. (2017). Bacterial metacommunity organization in a highly connected aquatic system. FEMS Microbiology Ecology, 93, 1–9. 10.1093/femsec/fiw225 27810879

[ece38091-bib-0026] Lee, S. H. , Sorensen, J. W. , Grady, K. L. , Tobin, T. C. , & Shade, A. (2017). Divergent extremes but convergent recovery of bacterial and archaeal soil communities to an ongoing subterranean coal mine fire. ISME Journal, 11, 1447–1459. 10.1038/ismej.2017.1 PMC5437352 28282042

[ece38091-bib-0027] MacArthur, R. H. (1957). On the relative abundance of bird species. Proceedings of the National Academy of Sciences of the United States of America, 43, 293–295. 10.1073/pnas.43.3.293 16590018 PMC528435

[ece38091-bib-0028] McNutt, M. K. , Camilli, R. , Crone, T. J. , Guthrie, G. D. , Hsieh, P. A. , Ryerson, T. B. , Savas, O. , & Shaffer, F. (2012). Review of flow rate estimates of the Deepwater Horizon oil spill. Proceedings of the National Academy of Sciences of the United States of America, 109(50), 20260–20267. 10.1073/pnas.1112139108 22187459 PMC3528583

[ece38091-bib-0029] Miller, J. I. , Techtmann, S. , Joyner, D. , Mahmoudi, N. , Fortney, J. , Fordyce, J. A. , GaraJayeva, N. , Askerov, F. S. , Cravid, C. , Kuijper, M. , Pelz, O. , & Hazen, T. C. (2020). Microbial communities across global marine basins show important compositional similarities by depth. MBio, 11, 1–12. 10.1128/mBio.01448-20 PMC7439485 32817104

[ece38091-bib-0030] Modin, O. , Liébana, R. , Saheb‐Alam, S. , Wilén, B. M. , Suarez, C. , Hermansson, M. , & Persson, F. (2020). Hill‐based dissimilarity indices and null models for analysis of microbial community assembly. Microbiome, 8, 132. 32917275 10.1186/s40168-020-00909-7 PMC7488682

[ece38091-bib-0031] National Research Council of the National Academies (2005). Oil spill dispersants: efficacy and effects. The National Academies Press.

[ece38091-bib-0032] Nikolova, C. , Ijaz, U. Z. , Magill, C. , Kleindienst, S. , Joye, S. , & Gutierrez, T. (2020). Response and oil degradation activities of a northeast Atlantic bacterial community to biogenic and synthetic surfactants. bioRxiv, 2020.12.18.423525. 10.1101/2020.12.18.423525 PMC8456599 34548108

[ece38091-bib-0033] Ning, D. , Deng, Y. , Tiedje, J. M. , & Zhou, J. (2019). A general framework for quantitatively assessing ecological stochasticity. Proceedings of the National Academy of Sciences, 116, 16892–16898. 10.1073/pnas.1904623116 PMC6708315 31391302

[ece38091-bib-0034] Presley, S. J. , Higgins, C. L. , & Willig, M. R. (2010). A comprehensive framework for the evaluation of metacommunity structure. Oikos, 119, 908–917. 10.1111/j.1600-0706.2010.18544.x

[ece38091-bib-0035] Rahsepar, S. , Smit, M. P. J. , Murk, A. J. , Rijnaarts, H. H. M. , & Langenhoff, A. A. M. (2016). Chemical dispersants: Oil biodegradation friend or foe? Marine Pollution Bulletin, 108, 113–119. 10.1016/j.marpolbul.2016.04.044 27156037

[ece38091-bib-0036] Redmond, M. , & Valentine, D. (2012). Natural gas and temperature structured a microbial community response to the Deepwater Horizon oil spill. Proceedings of the National Academy of Sciences, 109, 20292–20297. 10.1073/pnas.1108756108 PMC3528494 21969552

[ece38091-bib-0037] Rughöft, S. , Vogel, A. L. , Joye, S. B. , Gutierrez, T. , & Kleindienst, S. (2020). Starvation‐dependent inhibition of the hydrocarbon degrader marinobacter sp. TT1 by a chemical dispersant. Journal of Marine Science and Engineering, 8, 1–10. 10.3390/jmse8110925

[ece38091-bib-0038] Sale, P. F. (1979). Recruitment, loss and coexistence in a guild of territorial coral reef fishes. Oecologia, 42, 159–177. 10.1007/BF00344855 28309658

[ece38091-bib-0039] Stegen, J. C. , Lin, X. , Fredrickson, J. K. , Chen, X. , Kennedy, D. W. , Murray, C. J. , Rockhold, M. L. , & Konopka, A. (2013). Quantifying community assembly processes and identifying features that impose them. ISME Journal, 7, 2069–2079. 10.1038/ismej.2013.93 PMC3806266 23739053

[ece38091-bib-0040] Stegen, J. C. , Lin, X. , Fredrickson, J. K. , & Konopka, A. E. (2015a). Estimating and mapping ecological processes influencing microbial community assembly. Frontiers in Microbiology, 6, 370. 10.3389/fmicb.2015.00370 25983725 PMC4416444

[ece38091-bib-0041] Stegen, J. C. , Lin, X. , Fredrickson, J. K. , & Konopka, A. E. (2015b). Estimating and mapping ecological processes influencing microbial community assembly. Frontiers in Microbiology, 6, 370. 10.3389/fmicb.2015.00370 25983725 PMC4416444

[ece38091-bib-0042] Thomas, G. E. , Brant, J. L. , Campo, P. , Clark, D. R. , Coulon, F. , Gregson, B. H. , McGenity, T. J. , & McKew, B. A. (2021). Effects of Dispersants and Biosurfactants on Crude‐Oil Biodegradation and Bacterial Community Succession. Microorganisms, 9, 1200. 34206054 10.3390/microorganisms9061200 PMC8229435

[ece38091-bib-0043] Tillett, D. , & Neilan, B. A. (2000). Xanthogenate nucleic acid isolation from cultured and environmental cyanobacteria. Journal of Phycology, 36, 251–258. 10.1046/j.1529-8817.2000.99079.x

[ece38091-bib-0044] Trego, A. C. , McAteer, P. G. , Nzeteu, C. , Mahony, T. , Abram, F. , Ijaz, U. Z. , & O’Flaherty, V. (2021). Combined stochastic and deterministic processes drive community assembly of anaerobic microbiomes during granule flotation. Frontiers in Microbiology, 12, 1165. 10.3389/fmicb.2021.666584 PMC8160314 34054772

[ece38091-bib-0045] Tucker, C. M. , Shoemaker, L. G. , Davies, K. F. , Nemergut, D. R. , & Melbourne, B. A. (2016). Differentiating between niche and neutral assembly in metacommunities using null models of β‐diversity. Oikos, 125, 778–789. 10.1111/oik.02803

[ece38091-bib-0046] Vass, M. , Székely, A. J. , Lindström, E. S. , & Langenheder, S. (2020). Using null models to compare bacterial and microeukaryotic metacommunity assembly under shifting environmental conditions. Scientific Reports, 10, 1–13. 10.1038/s41598-020-59182-1 32051469 PMC7016149

[ece38091-bib-0047] Vellend, M. (2010). Conseptual synthesis in community ecology. The Quarterly Review of Biology, 85, 183–206. 20565040 10.1086/652373

[ece38091-bib-0048] Verster, A. J. , & Borenstein, E. (2018). Competitive lottery‐based assembly of selected clades in the human gut microbiome. Microbiome, 6, 186. 10.1186/s40168-018-0571-8 30340536 PMC6195700

[ece38091-bib-0049] Wu, W. , Lu, H.‐P. , Sastri, A. , Yeh, Y.‐C. , Gong, G.‐C. , Chou, W.‐C. , & Hsieh, C.‐H. (2018). Contrasting the relative importance of species sorting and dispersal limitation in shaping marine bacterial versus protist communities. ISME Journal, 12, 485–494. 10.1038/ismej.2017.183 PMC5776463 29125596

[ece38091-bib-0050] Zhou, J. , Deng, Y. E. , Zhang, P. , Xue, K. , Liang, Y. , Van Nostrand, J. D. , Yang, Y. , He, Z. , Wu, L. , Stahl, D. A. , Hazen, T. C. , Tiedje, J. M. , & Arkin, A. P. (2014). Stochasticity, succession, and environmental perturbations in a fluidic ecosystem. Proceedings of the National Academy of Sciences, 111, E836–E845. 10.1073/pnas.1324044111 PMC3948316 24550501

[ece38091-bib-0051] Zhou, J. , & Ning, D. (2017). Stochastic community assembly: Does it matter in microbial ecology? Microbiology and Molecular Biology Reviews, 81(4). 10.1128/MMBR.00002-17 PMC5706748 29021219

