# Supplementary figures and images for: Exploration of marine bacterioplankton community assembly mechanisms during chemical dispersant and surfactant‐assisted oil biodegradation
Source: Ecol Evol. 2021 Sep 10;11(20):13862–74. doi: 10.1002/ece3.8091 (PMC8525123; doi:10.1002/ece3.8091)

# Supplementary Table S1

**Supplementary Figure S1.**

**Supplementary Figure S2.**

**
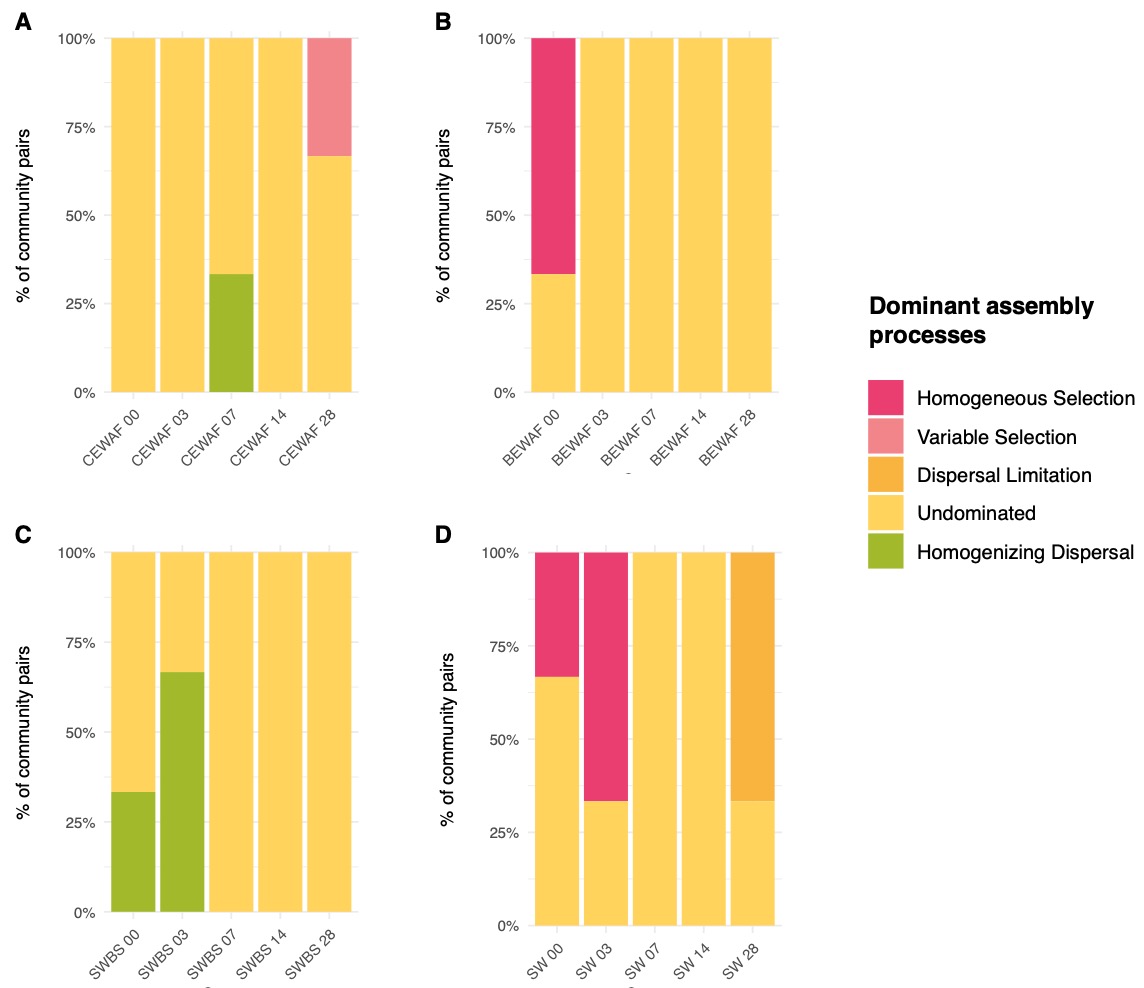
**

**Supplementary Figure S3**

Supplement: Supplementary file 1 — Supplementary Material [file ECE3-11-13862-s002.docx]
