# Supplementary material for: Exploration of marine bacterioplankton community assembly mechanisms during chemical dispersant and surfactant‐assisted oil biodegradation
Source: Ecol Evol. 2021 Sep 10;11(20):13862–74. doi: 10.1002/ece3.8091 (PMC8525123; doi:10.1002/ece3.8091)
Supplement: Supplementary file 2 — Appendix S1 [file ECE3-11-13862-s001.pdf]

Supplementary Results and Discussion

Results

Bacterial diversity variations

Similar to beta diversity analysis, the Local Contribution to Beta Diversity (LCBD) showed each sample's contribution to the observed changes derived as a proportion of the total beta diversity. The diversity patterns with high LCBD values suggested that those samples have markedly different microbial communities. Based on diversity counts (Bray-Curtis, Fig. S2), diversity variation generally increased over time across treatments. At day 0, all treatments show significantly lower, but similar diversity compared to the *in situ* FSC community. As time progressed, the within treatment diversity began to vary and at the end of the incubation period, the CEWAF and SWD treatments displayed the highest contribution to beta diversity variation, and hence their bacterial communities were significantly different from other treatments. Based on unweighted UniFrac, the community of the SWD treatment was significantly distinct from the other treatments, especially on day 7, which coincided with the emergent dominance of members within the family *Rhodobacteraceae*, such as of *Pseudophaeobacter*, *Sedimentitalea* and *Shimia*. In contrast, BEWAF and WAF treatments were dominated by members of the *Colwelliaceae* and *Saccharospirillaceae*.

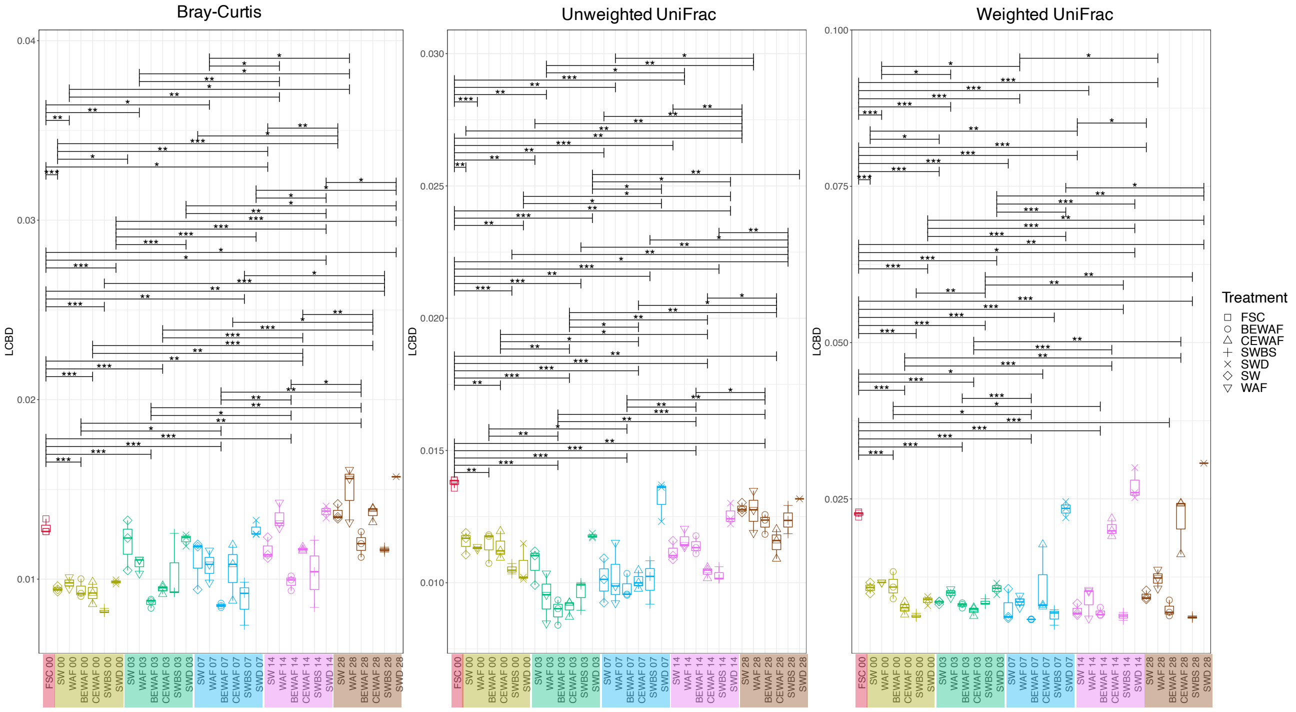

Figure S2. Local contribution to beta diversity (LCBD) for Bray-Curits, Unweighted and Weighted UniFrac distance matrices. Colours represent incubation time: red – in situ FSC at time of sampling, olive green – day 0, green – day 3, blue – day 7, pink – day 14 and brown – day 28. Statistically different treatments (pair-wise ANOVA) are annotated with bracket and the level of significance is shown above the bracket with a star sign: * is p<0.05, ** is p<0.01, and *** is p<0.001.

Regression analysis through supervised machine learning showed which variable best explained the variation of diversity in the dataset (Fig. S3). The variables that most significantly affected the alpha and beta diversity variation in the optimal model (with the lowest prediction error) are highlighted in red and blue. The top 10 predictive models for each metric of microbiome analysis – alpha diversity indices (Richness and Shannon), beta diversity (local contribution to beta diversity based on Bray-Curtis, Unweighted and Weighted UniFrac distance matrices), and NTI and NRI, were compiled into tables (Tables S1-7). The variables that positively affected the species richness and Shannon index was in situ FSC and incubation day 0, while incubation days 3 and 7, and treatments CEWAF and SWD, negatively affected alpha diversity variation. The treatments CEWAF, SWD, WAF and BEWAF, as well as incubation day 3, positively affected NRI variation, indicating that these treatments increased clustering of the bacterial communities. Conversely, no clustering was expected on incubation day 0. The presence of Finasol in the seawater (SWD treatment) positively influenced (or increased) the variation in beta diversity (LCBD) of the bacterial community. In terms of alpha

diversity, Finasol-amended treatments (CEWAF and SWD) reduced the species richness of the bacterial community. This coincided with the dominance of predominantly four taxa, *Colwellia*, *Oleispira*, *Sedimentitalea* and *Vibrio* (Figure 2 in main text) during the same period. In contrast, the *in situ* FSC community, which was not amended with crude oil, Finasol or the biosurfactant was a positively influencing parameter on alpha and beta diversity. The addition of crude oil alone to seawater (WAF) significantly influenced the variation of diversity in the dataset (LCBD based on Bray-Curtis) as well as the environmental filtering (NTI).

	Richness	Shannon	NRI	NTI	LCBD Bray-Curtis	LCBD Unweighted UniFrac	LCBD Weighted UniFrac
Treatment_CEWAF	***		***		***		***
Treatment_SWD	***	**	***	*	***	***	***
Treatment_BEWAF		**	***				
Treatment_SWBS							
Treatment_SW				*	***	**	
Treatment_WAF			*		***		
Treatment_FSC	***			**	***	***	***
Incubation_day_0	***	***	***		***		
Incubation_day_3	***	**	**			***	
Incubation_day_7	***			**			
Incubation_day_14					***		**
Incubation_day_28					***	***	***

Figure S3. Heatmap of significant extraneous parameters that influence different attributes of microbiome determined by regression analysis. The parameters shown here are from the optimal model for each metric, where blue represent significantly negative and red – significantly positive beta coefficients.

Key taxa representing major shift in the communities

To identify key taxa representing major shifts in the communities across the different treatments, differential abundance analysis was performed and the returned ASVs were an order of magnitude different between treatments. A larger log2 fold change is indicative of enrichment within each group. Common oil-degraders from the genera *Marinobacter*, *Oleispira*, and *Pseudomonas* were enriched in all treatments. *Vibrio*, *Oleiphilus,* and *Glaciecola* were enriched exclusively in CEWAF, while *Alcanivorax*, *Colwellia* and *Thalassotalea* (of the family *Colwelliaceae*) were enriched in both CEWAF and WAF. *Cycloclasticus*, *Pseudohongiella,* and *Acinetobacter* were enriched in the BEWAF and WAF

treatments, whereas *Alteromonas*, *Moritella* and *Paraglaciecola* were enriched exclusively in the BEWAF (Supplementary File 5).

Discussion

Impact on microbial diversity

We observed lower species richness (alpha diversity) in treatments with added dispersant (CEWAF and SWD) compared to the BEWAF and WAF treatments. For the CEWAF and SWD treatments, this can be explained by the bloom of a few specialist taxa in the first seven days of incubation, which had responded directly to the dispersed crude oil or the dispersant itself. To further confirm the main factors that significantly altered the species richness and beta diversity in our treatments, we performed supervised machine learning modelling (regression analysis). The regression analysis showed that the presence of Finasol indeed negatively affected the species richness of the FSC bacterial community, and more so than exposure by the oil or it also with the rhamnolipid. If two biological systems share many characteristics (i.e. temperature, depth, nutrient availability, species etc.), data generated in one system can help inform what changes can be expected in another system for which limited data exist. While conventional analyses on microbiomes focus more on the variability of community structure in view of extrinsic environmental parameters, the regression analyses, on the other hand, can offer directionality (positive/negative contribution) to diversity analyses, and are widely useful to delineate diversity changes in multitude of environments. To our knowledge, supervised machine learning modelling has rarely been used in marine microbial metagenomic studies, especially in the context of hydrocarbon pollution and synthetic dispersant application. However, examples of using regression analyses in other microbial community studies exist [1]. It was interesting to note that the addition of crude oil alone (WAF) did not affect the alpha and beta diversity significantly, indicating that the microbial community in this treatment maintained a higher diversity over time.

Table S1 Regression analysis top 10 models for Richness. The parameters of the model with the lowest Cross-validation error are given afterwards with significant positive influencers in red and negative in blue.

	Model	Cross-validation Errors
M8	Richness ~ Treatment_BEWAF + Treatment_CEWAF + Treatment_SWD + Treatment_SWBS + Treatment_FSC + Incubation_day_0 + Incubation_day_3 + Incubation_day_7	33.60742
M7	Richness ~ Treatment_BEWAF + Treatment_CEWAF + Treatment_SWD + Treatment_FSC + Incubation_day_0 + Incubation_day_3 + Incubation_day_7	33.70579
M6	Richness ~ Treatment_CEWAF + Treatment_SWD + Treatment_FSC + Incubation_day_0 + Incubation_day_3 + Incubation_day_7	34.72055
M9	Richness ~ Treatment_BEWAF + Treatment_CEWAF + Treatment_SWD + Treatment_SWBS + Treatment_FSC + Incubation_day_0 + Incubation_day_7 + Incubation_day_14 + Incubation_day_28	34.76996
M10	Richness ~ Treatment_WAF + Treatment_BEWAF + Treatment_CEWAF + Treatment_SWD + Treatment_SWBS + Treatment_FSC + Incubation_day_3 + Incubation_day_7 + Incubation_day_14 + Incubation_day_28	35.12272
M5	Richness ~ Treatment_CEWAF + Treatment_SWD + Incubation_day_0 + Incubation_day_3 + Incubation_day_7	37.53631
M4	Richness ~ Treatment_CEWAF + Treatment_SWD + Incubation_day_0 + Incubation_day_3	40.08586
M3	Richness ~ Treatment_SWD + Incubation_day_0 + Incubation_day_3	43.45814
M2	Richness ~ Treatment_SWD + Incubation_day_0	47.17362
M1	Richness ~ Incubation_day_0	52.23869

Richness M8							
**Predictors**	***Estimates***	***std. Error***	***std. Beta***	***CI***	***standardized CI***	***Statistic***	***p***
(Intercept)	180.69595 ***	7.59715		165.58000 – 195.81190		23.78470	<0.001
Treatment_BEWAF	20.84874	10.68310	0.10368	-0.40728 – 42.10476	-0.00045 – 0.20781	1.95156	0.054
Treatment_CEWAF	-39.09203 ***	10.68310	-0.19441	-60.34805 – -17.83600	-0.29854 – -0.09028	-3.65924	<0.001
Treatment_SWD	-66.88116 ***	11.23861	-0.31374	-89.24246 – -44.51985	-0.41708 – -0.21041	-5.95102	<0.001
Treatment_SWBS	-18.26529	10.68310	-0.09084	-39.52131 – 2.99074	-0.19497 – 0.01329	-1.70974	0.091
Treatment_FSC	79.71637 ***	21.71171	0.19095	36.51688 – 122.91587	0.08902 – 0.29289	3.67158	<0.001
Incubation_day_0	92.50323 ***	9.99698	0.51319	72.61238 – 112.39409	0.40449 – 0.62189	9.25312	<0.001
Incubation_day_3	-62.37200 ***	9.80121	-0.33293	-81.87332 – -42.87067	-0.43546 – -0.23039	-6.36371	<0.001
Incubation_day_7	-38.28133 ***	9.80121	-0.20434	-57.78265 – -18.78000	-0.30687 – -0.10180	-3.90578	<0.001
**Observations**	90						
**R^2 / R^2 adjusted**	0.819 / 0.801						
							*** p<0.05 ** p<0.01 *** p<0.001**

Table S2 Regression analysis top 10 models for Shannon index. The parameters of the model with the lowest Cross-validation error are given afterwards with significant positive influencers in red and negative in blue.

	Model	Cross-validation Errors
M6	Shannon ~ Treatment_BEWAF + Treatment_SWD + Treatment_SWBS + Incubation_day_0 + Incubation_day_3 + Incubation_day_28	0.52981
M5	Shannon ~ Treatment_BEWAF + Treatment_SWD + Incubation_day_0 + Incubation_day_3 + Incubation_day_28	0.53067
M4	Shannon ~ Treatment_BEWAF + Treatment_SWD + Incubation_day_0 + Incubation_day_3	0.53191
M7	Shannon ~ Treatment_BEWAF + Treatment_SWD + Treatment_SWBS + Incubation_day_0 + Incubation_day_3 + Incubation_day_7 + Incubation_day_28	0.53729
M9	Shannon ~ Treatment_BEWAF + Treatment_CEWAF + Treatment_SWD + Treatment_SWBS + Treatment_SW + Incubation_day_0 + Incubation_day_3 + Incubation_day_7 + Incubation_day_14	0.53944
M8	Shannon ~ Treatment_BEWAF + Treatment_CEWAF + Treatment_SWD + Treatment_SWBS + Incubation_day_3 + Incubation_day_7 + Incubation_day_14 + Incubation_day_28	0.53996
M10	Shannon ~ Treatment_WAF + Treatment_BEWAF + Treatment_CEWAF + Treatment_SWD + Treatment_SWBS + Treatment_SW + Incubation_day_0 + Incubation_day_3 + Incubation_day_7 + Incubation_day_14	0.54886
M3	Shannon ~ Treatment_SWD + Incubation_day_0 + Incubation_day_3	0.55250
M2	Shannon ~ Treatment_SWD + Incubation_day_0	0.57346
M1	Shannon ~ Incubation_day_0	0.62367

Shannon M6							
Predictors	*Estimates*	*std. Error*	*std. Beta*	*CI*	*standardized CI*	*Statistic*	*p*
(Intercept)	2.75576 ***	0.10417		2.54856 – 2.96296		26.45324	<0.001
Treatment_BEWAF	0.48462 **	0.15655	0.21540	0.17325 – 0.79598	0.07902 – 0.35178	3.09562	0.003
Treatment_SWD	-0.53318 **	0.16627	-0.22355	-0.86389 – -0.20248	-0.36018 – -0.08691	-3.20670	0.002
Treatment_SWBS	0.16425	0.15655	0.07301	-0.14712 – 0.47562	-0.06337 – 0.20939	1.04920	0.297
Incubation_day_0	1.31226 ***	0.14727	0.65067	1.01936 – 1.60517	0.50755 – 0.79379	8.91081	<0.001
Incubation_day_3	-0.41187 **	0.15233	-0.19649	-0.71484 – -0.10890	-0.33892 – -0.05406	-2.70389	0.008
Incubation_day_28	0.25684	0.15941	0.11712	-0.06022 – 0.57390	-0.02535 – 0.25958	1.61119	0.111
**Observations**	90						
**R^2 / R^2 adjusted**	0.635 / 0.608						
							*** p<0.05 ** p<0.01 *** p<0.001**

Table S3 Regression analysis top 10 models for NTI. The parameters of the model with the lowest Cross-validation error are given afterwards with significant positive influencers in red and negative in blue.

	Model	Cross-validation Errors
**M6**	NTI ~ Treatment_CEWAF + Treatment_SWD + Treatment_SW + Treatment_FSC + Incubation_day_7 + Incubation_day_14	0.98634
**M7**	NTI ~ Treatment_CEWAF + Treatment_SWD + Treatment_SWBS + Treatment_SW + Treatment_FSC + Incubation_day_7 + Incubation_day_14	0.98841
**M8**	NTI ~ Treatment_CEWAF + Treatment_SWD + Treatment_SWBS + Treatment_SW + Treatment_FSC + Incubation_day_0 + Incubation_day_7 + Incubation_day_14	0.98919
**M5**	NTI ~ Treatment_SWD + Treatment_SW + Treatment_FSC + Incubation_day_7 + Incubation_day_14	0.99991
**M9**	NTI ~ Treatment_WAF + Treatment_BEWAF + Treatment_CEWAF + Treatment_SWBS + Treatment_FSC + Incubation_day_3 + Incubation_day_7 + Incubation_day_14 + Incubation_day_28	1.00014
**M4**	NTI ~ Treatment_SWD + Treatment_SW + Treatment_FSC + Incubation_day_7	1.00617
**M10**	NTI ~ Treatment_WAF + Treatment_CEWAF + Treatment_SWD + Treatment_SWBS + Treatment_SW + Treatment_FSC + Incubation_day_0 + Incubation_day_3 + Incubation_day_7 + Incubation_day_28	1.01131
**M3**	NTI ~ Treatment_CEWAF + Treatment_FSC + Incubation_day_7	1.01589
**M2**	NTI ~ Treatment_FSC + Incubation_day_7	1.05334
**M1**	NTI ~ Treatment_FSC	1.08783

**NTI M6**							
Predictors	*Estimates*	*std. Error*	*std. Beta*	*CI*	*standardized CI*	*Statistic*	*p*
(Intercept)	6.58923 ***	0.17336		6.24443 – 6.93402		38.00993	<0.001
Treatment_CEWAF	0.48364	0.29596	0.15687	-0.10501 – 1.07230	-0.03128 – 0.34501	1.63414	0.106
Treatment_SWD	-0.64640 *	0.31268	-0.19776	-1.26830 – -0.02450	-0.38526 – -0.01027	-2.06730	0.042
Treatment_SW	-0.69154 *	0.29596	-0.22430	-1.28020 – -0.10289	-0.41244 – -0.03615	-2.33659	0.022
Treatment_FSC	-1.90112 **	0.59721	-0.29700	-3.08894 – -0.71329	-0.47986 – -0.11414	-3.18334	0.002
Incubation_day_7	0.88635 **	0.27153	0.30856	0.34630 – 1.42640	0.12329 – 0.49382	3.26433	0.002
Incubation_day_14	0.46463	0.27153	0.16175	-0.07542 – 1.00469	-0.02352 – 0.34701	1.71119	0.091
**Observations**	90						
**R^2 / R^2 adjusted**	0.316 / 0.266						
							*** p<0.05 ** p<0.01 *** p<0.001**

Table S4 Regression analysis top 10 models for NRI. The parameters of the model with the lowest Cross-validation error are given afterwards with significant positive influencers in red and negative in blue.

	Model	Cross-validation Errors
**M8**	NRI ~ Treatment_WAF + Treatment_BEWAF + Treatment_CEWAF + Treatment_SWD + Treatment_SWBS + Incubation_day_0 + Incubation_day_3 + Incubation_day_14	1.11824
**M7**	NRI ~ Treatment_BEWAF + Treatment_CEWAF + Treatment_SWD + Treatment_SW + Incubation_day_0 + Incubation_day_3 + Incubation_day_14	1.11853
**M9**	NRI ~ Treatment_WAF + Treatment_BEWAF + Treatment_CEWAF + Treatment_SWD + Treatment_SWBS + Treatment_SW + Incubation_day_0 + Incubation_day_3 + Incubation_day_14	1.12987
**M6**	NRI ~ Treatment_BEWAF + Treatment_CEWAF + Treatment_SWD + Treatment_SW + Incubation_day_0 + Incubation_day_3	1.13572
**M5**	NRI ~ Treatment_BEWAF + Treatment_CEWAF + Treatment_SWD + Incubation_day_0 + Incubation_day_3	1.14052
**M10**	NRI ~ Treatment_WAF + Treatment_BEWAF + Treatment_CEWAF + Treatment_SWD + Treatment_SWBS + Treatment_FSC + Incubation_day_3 + Incubation_day_7 + Incubation_day_14 + Incubation_day_28	1.14529
**M4**	NRI ~ Treatment_CEWAF + Treatment_SWD + Incubation_day_0 + Incubation_day_3	1.19900
**M3**	NRI ~ Treatment_CEWAF + Treatment_SWD + Incubation_day_0	1.27369
**M2**	NRI ~ Treatment_SWD + Incubation_day_0	1.54889
**M1**	NRI ~ Incubation_day_0	1.70878

**NRI M8**							
Predictors	*Estimates*	*std. Error*	*std. Beta*	*CI*	*standardized CI*	*Statistic*	*p*
(Intercept)	1.81273 ***	0.29594		1.22390 – 2.40157		6.12525	<0.001
Treatment_WAF	0.88162 *	0.38237	0.14220	0.12083 – 1.64241	0.02132 – 0.26308	2.30570	0.024
Treatment_BEWAF	1.61687 ***	0.37343	0.26817	0.87387 – 2.35988	0.14678 – 0.38956	4.32983	<0.001
Treatment_CEWAF	3.26464 ***	0.37343	0.54146	2.52164 – 4.00764	0.42007 – 0.66285	8.74239	<0.001
Treatment_SWD	3.45907 ***	0.38842	0.54117	2.68624 – 4.23190	0.42206 – 0.66027	8.90555	<0.001
Treatment_SWBS	0.72814	0.37343	0.12077	-0.01486 – 1.47114	-0.00062 – 0.24216	1.94988	0.055
Incubation_day_0	-3.28898 ***	0.30243	-0.60853	-3.89072 – -2.68725	-0.71820 – -0.49886	-10.87535	<0.001
Incubation_day_3	0.85700 **	0.31027	0.15256	0.23966 – 1.47435	0.04430 – 0.26082	2.76209	0.007
Incubation_day_14	-0.56575	0.31027	-0.10071	-1.18310 – 0.05160	-0.20897 – 0.00754	-1.82339	0.072
**Observations**	90						
**R^2 / R^2 adjusted**	0.799 / 0.779						
							*** p<0.05 ** p<0.01 *** p<0.001**

Table S5 Regression analysis top 10 models for LCBD (Bray-Curtis). The parameters of the model with the lowest Cross-validation error are given afterwards with significant positive influencers in red and negative in blue.

	Model	Cross-validation Errors
M8	LCBD ~ Treatment_WAF + Treatment_CEWAF + Treatment_SWD + Treatment_SW + Treatment_FSC + Incubation_day_0 + Incubation_day_14 + Incubation_day_28	0.00102
M9	LCBD ~ Treatment_WAF + Treatment_CEWAF + Treatment_SWD + Treatment_SW + Treatment_FSC + Incubation_day_0 + Incubation_day_3 + Incubation_day_7 + Incubation_day_28	0.00103
M10	LCBD ~ Treatment_WAF + Treatment_BEWAF + Treatment_CEWAF + Treatment_SWD + Treatment_SWBS + Treatment_SW + Incubation_day_0 + Incubation_day_3 + Incubation_day_7 + Incubation_day_28	0.00106
M7	LCBD ~ Treatment_BEWAF + Treatment_SWD + Treatment_SWBS + Treatment_FSC + Incubation_day_0 + Incubation_day_14 + Incubation_day_28	0.00108
M6	LCBD ~ Treatment_BEWAF + Treatment_SWD + Treatment_SWBS + Treatment_FSC + Incubation_day_14 + Incubation_day_28	0.00115
M5	LCBD ~ Treatment_BEWAF + Treatment_CEWAF + Treatment_SWBS + Incubation_day_14 + Incubation_day_28	0.00119
M4	LCBD ~ Treatment_BEWAF + Treatment_SWBS + Incubation_day_14 + Incubation_day_28	0.00127
M3	LCBD ~ Treatment_BEWAF + Treatment_SWBS + Incubation_day_28	0.00138
M2	LCBD ~ Treatment_BEWAF + Incubation_day_28	0.00157
M1	LCBD ~ Incubation_day_28	0.00169

LCBD Bray-Curtis M8							
Predictors	*Estimates*	*std. Error*	*std. Beta*	*CI*	*standardized CI*	*Statistic*	*p*
(Intercept)	0.00915 ***	0.00022		0.00871 – 0.00959		41.47606	**<0.001**
Treatment_WAF	0.00219 ***	0.00032	0.40460	0.00156 – 0.00283	0.28952 – 0.51968	6.89090	**<0.001**
Treatment_SWD	0.00301 ***	0.00033	0.53837	0.00236 – 0.00366	0.42338 – 0.65335	9.17646	**0.001**
Treatment_CEWAF	0.00112 ***	0.00031	0.21336	0.00051 – 0.00174	0.09789 – 0.32884	3.62132	**<0.001**
Treatment_SW	0.00178 ***	0.00031	0.33850	0.00117 – 0.00240	0.22302 – 0.45398	5.74523	**<0.001**
Treatment_FSC	0.00489 ***	0.00063	0.44685	0.00363 – 0.00615	0.33372 – 0.55998	7.74154	**<0.001**
Incubation_day_0	-0.00115 ***	0.00029	-0.24326	-0.00172 – -0.00057	-0.36328 – -0.12323	-3.97233	**<0.001**
Incubation_day_14	0.00127 ***	0.00028	-0.25957	0.00071 – 0.00184	0.14642 – 0.37272	4.49640	**<0.001**
Incubation_day_28	0.00304 ***	0.00030	0.59274	0.00245 – 0.00364	0.47955 – 0.70592	10.26445	**<0.001**
**Observations**	90						
**R^2 / R^2 adjusted**	0.775 / 0.753						
							*** p<0.05 ** p<0.01 *** p<0.001**

Table S6 Regression analysis top 10 models for LCBD (unweighted UniFrac). The parameters of the model with the lowest Cross-validation error are given afterwards with significant positive influencers in red and negative in blue.

	Model	Cross-validation Errors
M7	LCBD ~ Treatment_WAF + Treatment_SWD + Treatment_SW + Treatment_FSC + Incubation_day_3 + Incubation_day_7 + Incubation_day_28	0.00083
M8	LCBD ~ Treatment_WAF + Treatment_BEWAF + Treatment_SWD + Treatment_SW + Treatment_FSC + Incubation_day_3 + Incubation_day_7 + Incubation_day_28	0.00083
M6	LCBD ~ Treatment_SWD + Treatment_SW + Treatment_FSC + Incubation_day_3 + Incubation_day_7 + Incubation_day_28	0.00084
M9	LCBD ~ Treatment_WAF + Treatment_BEWAF + Treatment_SWD + Treatment_SW + Treatment_FSC + Incubation_day_0 + Incubation_day_3 + Incubation_day_7 + Incubation_day_28	0.00084
M4	LCBD ~ Treatment_SWD + Treatment_FSC + Incubation_day_3 + Incubation_day_28	0.00085
M5	LCBD ~ Treatment_SWD + Treatment_FSC + Incubation_day_3 + Incubation_day_7 + Incubation_day_28	0.00085
M10	LCBD ~ Treatment_WAF + Treatment_BEWAF + Treatment_SWD + Treatment_SWBS + Treatment_SW + Treatment_FSC + Incubation_day_0 + Incubation_day_3 + Incubation_day_7 + Incubation_day_14	0.00085
M3	LCBD ~ Treatment_SWD + Treatment_FSC + Incubation_day_28	0.00093
M2	LCBD ~ Treatment_FSC + Incubation_day_28	0.00105
M1	LCBD ~ Incubation_day_28	0.00119

LCBD Unweighted UniFrac M7							
Predictors	*Estimates*	*std. Error*	*std. Beta*	*CI*	*standardized CI*	*Statistic*	*p*
(Intercept)	0.01071 ***	0.00015		0.01040 – 0.01101		70.10001	<0.001
Treatment_WAF	0.00044	0.00024	0.11977	-0.00003 – 0.00090	-0.00670 – 0.24623	1.85613	0.067
Treatment_SWD	0.00174 ***	0.00024	0.46183	0.00125 – 0.00222	0.33493 – 0.58873	7.13305	<0.001
Treatment_SW	0.00066 **	0.00023	0.18508	0.00020 – 0.00111	0.05845 – 0.31171	2.86468	0.005
Treatment_FSC	0.00307 ***	0.00047	0.41684	0.00214 – 0.00400	0.29192 – 0.54176	6.54018	<0.001
Incubation_day_3	-0.00125 ***	0.00022	-0.37971	-0.00170 – -0.00081	-0.51197 – -0.24745	-5.62684	<0.001
Incubation_day_7	-0.00060 **	0.00022	-0.18051	-0.00104 – -0.00015	-0.31277 – -0.04825	-2.67496	0.009
Incubation_day_28	0.00136 ***	0.00023	0.39479	0.00090 – 0.00183	0.26248 – 0.52710	5.84801	<0.001
**Observations**	90						
**R^2 / R^2 adjusted**	0.692 / 0.666						
							*** p<0.05 ** p<0.01 *** p<0.001**

Table S7 Regression analysis top 10 models for LCBD (weighted UniFrac). The parameters of the model with the lowest Cross-validation error are given afterwards with significant positive influencers in red and negative in blue.

	**Model**	**Cross-validation Errors**
M8	LCBD ~ Treatment_WAF + Treatment_CEWAF + Treatment_SWD + Treatment_SWBS + Treatment_FSC + Incubation_day_7 + Incubation_day_14 + Incubation_day_28	0.00449
M7	LCBD ~ Treatment_WAF + Treatment_CEWAF + Treatment_SWD + Treatment_FSC + Incubation_day_7 + Incubation_day_14 + Incubation_day_28	0.00450
M6	LCBD ~ Treatment_WAF + Treatment_CEWAF + Treatment_SWD + Treatment_FSC + Incubation_day_14 + Incubation_day_28	0.00454
M9	LCBD ~ Treatment_WAF + Treatment_BEWAF + Treatment_CEWAF + Treatment_SWD + Treatment_SWBS + Treatment_SW + Incubation_day_7 + Incubation_day_14 + Incubation_day_28	0.00455
M10	LCBD ~ Treatment_WAF + Treatment_BEWAF + Treatment_CEWAF + Treatment_SWBS + Treatment_SW + Treatment_FSC + Incubation_day_0 + Incubation_day_3 + Incubation_day_14 + Incubation_day_28	0.00455
M5	LCBD ~ Treatment_CEWAF + Treatment_SWD + Treatment_FSC + Incubation_day_14 + Incubation_day_28	0.00460
M4	LCBD ~ Treatment_CEWAF + Treatment_SWD + Treatment_FSC + Incubation_day_28	0.00461
M3	LCBD ~ Treatment_CEWAF + Treatment_SWD + Treatment_FSC	0.00481
M2	LCBD ~ Treatment_SWD + Treatment_FSC	0.00508
M1	LCBD ~ Treatment_SWD	0.00555

**LCBD Weighted UniFrac M8**							
Predictors	*Estimates*	*std. Error*	*std. Beta*	*CI*	*standardized CI*	*Statistic*	*p*
(Intercept)	0.00627 ***	0.00097		0.00434 – 0.00820		6.47096	<0.001
Treatment_WAF	0.00176	0.00139	0.10139	-0.00102 – 0.00453	-0.05630 – 0.25908	1.26016	0.211
Treatment_CEWAF	0.00529 ***	0.00136	0.31377	0.00258 – 0.00800	0.15542 – 0.47213	3.88362	<0.001
Treatment_SWD	0.01063 ***	0.00144	0.59516	0.00777 – 0.01349	0.43748 – 0.75284	7.39793	<0.001
Treatment_SWBS	-0.00157	0.00136	-0.09306	-0.00428 – 0.00114	-0.25142 – 0.06529	-1.15186	0.253
Treatment_FSC	0.01631 ***	0.00267	0.46608	0.01100 – 0.02162	0.31662 – 0.61554	6.11212	<0.001
Incubation_day_7	0.00149	0.00125	0.09473	-0.00100 – 0.00397	-0.06120 – 0.25067	1.19072	0.237
Incubation_day_14	0.00378 **	0.00125	0.24070	0.00129 – 0.00626	0.08476 – 0.39664	3.02537	0.003
Incubation_day_28	0.00459 ***	0.00131	0.27923	0.00199 – 0.00719	0.12323 – 0.43522	3.50822	0.001
**Observations**	90						
**R^2 / R^2 adjusted**	0.577 / 0.535						
							*** p<0.05 ** p<0.01 *** p<0.001**

Table S8. Statistical summary of all predicted pathways which were significantly different between treatments BEWAF, CEWAF, and WAF (ANOVA). Size means the total KEGG orthologs (KO) that are present in the particular pathway. Hits is the number of KEGG orthologs that were predicted in our data set. Pval is p-value based on ANOVA and FDR is the false discovery rate. The table was generated in Microbiomeanalyst online tool [2] using the KO abundance table generated in PICRUsT2.

Pathway	Size	Hits	Statistic.Q	Expected.Q	Pval	FDR
Carotenoid biosynthesis	27	15	9.97168466	1.13596366	1.94E-12	2.68E-10
Xylene degradation	20	18	5.28809519	1.13591273	4.16E-10	2.14E-08
Betalain biosynthesis	3	3	9.12897169	1.13595138	6.12E-10	2.14E-08
Indole alkaloid biosynthesis	3	1	9.13653965	1.13595062	6.19E-10	2.14E-08
Degradation of aromatic compounds	134	81	6.18544513	1.1361019	3.05E-09	8.41E-08
Styrene degradation	12	9	6.34546	1.13611384	1.17E-08	2.69E-07
Glycosylphosphatidylinositol(GPI)-anchor biosynthesis	17	1	5.40109822	1.13488291	2.81E-08	5.54E-07
Toluene degradation	35	24	4.40880673	1.13598138	1.32E-07	2.28E-06
Penicillin and cephalosporin biosynthesis	8	2	6.57054924	1.1356708	2.71E-07	4.16E-06
Dioxin degradation	25	18	4.15338553	1.13604411	5.49E-07	7.06E-06
Phosphonate and phosphinate metabolism	9	7	4.74904	1.13602909	5.63E-07	7.06E-06
Chlorocyclohexane and chlorobenzene degradation	37	22	5.18354963	1.13608511	1.42E-06	1.64E-05
Fatty acid elongation	9	4	4.11550624	1.13578324	3.71E-06	3.76E-05
Atrazine degradation	10	7	6.22756395	1.13610806	3.84E-06	3.76E-05
Ethylbenzene degradation	13	10	5.06309761	1.13593171	4.08E-06	3.76E-05
Polycyclic aromatic hydrocarbon degradation	30	11	5.3210803	1.13605253	4.48E-06	3.87E-05
Brassinosteroid biosynthesis	8	1	5.83073314	1.13599469	1.33E-05	0.00010813
Sphingolipid metabolism	29	9	5.38022467	1.1361375	1.72E-05	0.00013151
Nicotinate and nicotinamide metabolism	36	24	4.26285119	1.13616091	2.18E-05	0.00015039
Galactose metabolism	56	36	3.97106009	1.13616143	2.18E-05	0.00015039
Sulfur metabolism	38	33	4.35511156	1.1361461	2.89E-05	0.00018992
Steroid biosynthesis	26	12	2.6769289	1.13568296	3.85E-05	0.00024157
Phenylpropanoid biosynthesis	18	7	4.20373168	1.13615719	4.89E-05	0.00029369
Other glycan degradation	7	6	4.26344332	1.1361368	6.28E-05	0.00036083
Isoquinoline alkaloid biosynthesis	34	8	3.96347065	1.13616444	9.80E-05	0.00054072
Glycine, serine and threonine metabolism	78	68	3.9838976	1.13614447	0.00013135	0.00069716
Porphyrin and chlorophyll metabolism	69	61	3.78837438	1.13616769	0.00015668	0.00080079
Chloroalkane and chloroalkene degradation	23	18	4.43373845	1.13611423	0.00016906	0.00083323
Ubiquinone and other terpenoid-quinone biosynthesis	40	32	3.67440507	1.13616153	0.00018231	0.00086753

Pentose and glucuronate interconversions	41	29	4.34074766	1.13612532	0.00019185	0.00088252
Thiamine metabolism	23	19	3.64862103	1.13617186	0.00022895	0.00100231
Ascorbate and aldarate metabolism	31	21	4.59681155	1.13610289	0.00023242	0.00100231
Fructose and mannose metabolism	44	39	3.51012366	1.136164	0.00025099	0.0010496
alpha-Linolenic acid metabolism	18	3	4.61799267	1.13596199	0.00032437	0.00131654
Arginine and proline metabolism	115	87	3.77211405	1.13614983	0.00037629	0.00148366
Fluorobenzoate degradation	11	10	3.48659567	1.13601335	0.00043032	0.00164958
Glycerolipid metabolism	57	21	4.01485804	1.13613582	0.00044418	0.00165667
Histidine metabolism	35	29	3.87528775	1.13614082	0.00045897	0.00166678
Tyrosine metabolism	54	29	3.545479	1.13616393	0.00051483	0.00182172
Taurine and hypotaurine metabolism	17	14	3.34317642	1.1361523	0.00061986	0.00213851
Ether lipid metabolism	26	6	4.00333279	1.13607104	0.00066002	0.00222153
Limonene and pinene degradation	7	5	4.00686728	1.13611285	0.00068264	0.00224296
Arachidonic acid metabolism	42	2	4.41942989	1.1360507	0.00073724	0.00236603
Flavonoid biosynthesis	12	2	4.21685835	1.13584686	0.00081901	0.00256873
Cysteine and methionine metabolism	71	55	3.27883171	1.13615863	0.0008919	0.00273517
Folate biosynthesis	29	25	3.2721364	1.13616078	0.0009636	0.00289081
Selenocompound metabolism	15	13	3.43120091	1.13616047	0.00111601	0.0032768
Alanine, aspartate and glutamate metabolism	62	47	3.34549003	1.1361575	0.0012383	0.00356011
Benzoate degradation	59	46	3.37205583	1.1361471	0.00136426	0.0038422
Starch and sucrose metabolism	65	50	2.94813513	1.13616902	0.00153493	0.00423641
Methane metabolism	105	72	3.16793163	1.13616318	0.00158714	0.00429461
beta-Alanine metabolism	36	27	3.46033817	1.13612818	0.00176144	0.00467459
Pentose phosphate pathway	63	45	2.98081081	1.1361657	0.00187516	0.0048825
Drug metabolism - other enzymes	20	14	3.2831644	1.13614947	0.00200104	0.00511376
Steroid degradation	9	9	3.35937159	1.1360578	0.00221512	0.00555793
Naphthalene degradation	17	12	2.94100972	1.13613197	0.00225706	0.00556203
Butirosin and neomycin biosynthesis	3	2	3.88950241	1.13603624	0.00234952	0.00565521
Cyanoamino acid metabolism	10	7	3.23158042	1.13613768	0.00237683	0.00565521
Tetracycline biosynthesis	6	6	3.85238356	1.13607273	0.00247895	0.00579111
Lipopolysaccharide biosynthesis	17	17	3.48968071	1.13612783	0.002519	0.00579111
Glycolysis / Gluconeogenesis	80	62	3.1556835	1.13615494	0.00255984	0.00579111
Drug metabolism - cytochrome P450	19	5	2.94129609	1.13612488	0.00268369	0.00597337
Nitrotoluene degradation	7	7	3.4639434	1.13594147	0.00275845	0.00604232
Purine metabolism	181	118	3.1452387	1.13615205	0.00339601	0.00719095
One carbon pool by folate	24	18	3.28647671	1.13611624	0.00343905	0.00719095
Amino sugar and nucleotide sugar metabolism	64	52	3.01190304	1.13615415	0.00343915	0.00719095
Glycerophospholipid metabolism	65	28	2.99047739	1.13615929	0.00467197	0.00962286
Aminobenzoate degradation	20	13	2.62855792	1.13613028	0.00491588	0.00997635

Phenylalanine, tyrosine and tryptophan biosynthesis	64	50	2.93225118	1.13614178	0.00588982	0.01177965
Pyrimidine metabolism	149	92	3.00625875	1.13614791	0.0067611	0.01332903
Pyruvate metabolism	74	64	2.88508525	1.13615762	0.00726018	0.01411133
Steroid hormone biosynthesis	33	5	2.98297478	1.13593603	0.00761162	0.01458894
Glutathione metabolism	28	16	2.8467084	1.13614829	0.0098772	0.01841114
Sesquiterpenoid and triterpenoid biosynthesis	11	3	2.78815187	1.13518136	0.01023049	0.01841114
Lysine degradation	30	22	2.8702454	1.13614015	0.01023455	0.01841114
Glycosaminoglycan biosynthesis - chondroitin sulfate / dermatan sulfate	10	2	2.70286248	1.13561504	0.01035069	0.01841114
Glycosaminoglycan biosynthesis - heparan sulfate / heparin	9	2	2.70286248	1.13561504	0.01035069	0.01841114
Retinol metabolism	41	7	2.60807976	1.1361166	0.0105058	0.01841114
Metabolism of xenobiotics by cytochrome P450	21	4	2.60795655	1.13611644	0.01053971	0.01841114
Nitrogen metabolism	17	16	2.92958808	1.13613307	0.01067356	0.0184119
Inositol phosphate metabolism	39	13	2.77623819	1.13614249	0.01119923	0.01908017
Tryptophan metabolism	42	24	2.80704319	1.13613961	0.01152336	0.01939296
Carbapenem biosynthesis	2	2	3.08588892	1.13610558	0.01297213	0.02156811
Peptidoglycan biosynthesis	13	10	3.01916776	1.13611262	0.01422744	0.02337365
Carbon metabolism	249	177	2.55921016	1.13616162	0.01516148	0.0246151
Phenylalanine metabolism	41	25	2.45206162	1.13616579	0.01601087	0.02560782
Caprolactam degradation	13	11	2.34638479	1.13614838	0.01614406	0.02560782
Fatty acid degradation	39	24	2.60667707	1.13614359	0.01680699	0.02635641
Glycosaminoglycan degradation	14	10	2.65700099	1.13611735	0.01746633	0.02708263
Carbon fixation in photosynthetic organisms	35	24	2.55865784	1.13615138	0.01878063	0.02879696
Biosynthesis of amino acids	222	195	2.54177795	1.13615116	0.01920682	0.02885052
Valine, leucine and isoleucine degradation	58	41	2.57720101	1.13614163	0.01950582	0.02885052
Glyoxylate and dicarboxylate metabolism	51	41	2.53849201	1.13615598	0.01958665	0.02885052
D-Glutamine and D-glutamate metabolism	6	6	2.57760322	1.13614301	0.01965181	0.02885052
Novobiocin biosynthesis	12	9	2.40828911	1.13616563	0.02026353	0.02943544
Stilbenoid, diarylheptanoid and gingerol biosynthesis	5	2	2.71013396	1.13556871	0.02110049	0.03033196
Oxidative phosphorylation	16	10	2.7380459	1.1361017	0.02272129	0.03232514
Citrate cycle (TCA cycle)	53	38	2.52373591	1.13615915	0.02320847	0.03268132
Vitamin B6 metabolism	12	9	2.52235243	1.13614189	0.02357859	0.03274728
Pantothenate and CoA biosynthesis	29	24	2.55685307	1.13614003	0.02372992	0.03274728
Lysine biosynthesis	41	35	2.48442961	1.13615221	0.02406931	0.03288677
Geraniol degradation	6	6	2.17660482	1.1361301	0.02679093	0.03624656
Biosynthesis of siderophore group nonribosomal peptides	3	2	2.33099871	1.13600902	0.02914228	0.03904499
Glycosphingolipid biosynthesis - globo series	10	1	2.65051469	1.13609443	0.0323611	0.04253174

Glycosphingolipid biosynthesis - ganglio series	13	1	2.65051469	1.13609443	0.0323611	0.04253174
Caffeine metabolism	9	3	2.44282322	1.13549754	0.03342054	0.04350976
Various types of N-glycan biosynthesis	28	4	2.50795012	1.13593671	0.0353728	0.04562099
Carbon fixation pathways in prokaryotes	60	49	2.29028591	1.1361594	0.03849426	0.04918711
Fatty acid biosynthesis	28	23	2.31768643	1.13614242	0.04036355	0.05067933
Linoleic acid metabolism	20	3	2.19093346	1.13616274	0.04039657	0.05067933
2-Oxocarboxylic acid metabolism	57	44	2.27845284	1.1361506	0.04155126	0.05165833
Primary bile acid biosynthesis	14	3	2.24930887	1.13610288	0.04376648	0.05392655
C5-Branched dibasic acid metabolism	11	11	2.33328086	1.1361248	0.04762938	0.05816686
Flavone and flavonol biosynthesis	3	1	2.43506139	1.13608911	0.04868892	0.05893921
Biotin metabolism	19	17	2.18432575	1.13614387	0.05213289	0.06255947
Riboflavin metabolism	22	14	2.22311294	1.1361467	0.05386956	0.0640862
D-Alanine metabolism	3	3	2.27691485	1.13614682	0.05485247	0.06469778
Tropane, piperidine and pyridine alkaloid biosynthesis	19	9	2.0954149	1.13616168	0.05804609	0.06788441
Lipoic acid metabolism	4	3	2.27358553	1.13613219	0.06028646	0.06991203
Butanoate metabolism	61	56	2.09161276	1.1361548	0.06134395	0.07033526
Streptomycin biosynthesis	12	11	2.06774132	1.13615673	0.06167077	0.07033526
Valine, leucine and isoleucine biosynthesis	15	15	2.1316621	1.13612152	0.07133274	0.08068786
Zeatin biosynthesis	3	1	2.22186941	1.13609808	0.07256124	0.08141017
Fatty acid metabolism	52	38	2.01047677	1.13615393	0.07396626	0.08231729
Propanoate metabolism	55	47	1.99682166	1.13615136	0.07582248	0.08370801
Biosynthesis of vancomycin group antibiotics	1	1	2.07213059	1.136114	0.09530025	0.10437646
Biosynthesis of ansamycins	1	1	2.0412	1.13610372	0.10045036	0.10915078
Bisphenol degradation	6	4	1.83627024	1.13606084	0.1306644	0.14087256
Other types of O-glycan biosynthesis	11	1	1.73995698	1.134569	0.13605017	0.14332003
Glycosaminoglycan biosynthesis - keratan sulfate	9	1	1.73995698	1.134569	0.13605017	0.14332003
Glycosphingolipid biosynthesis - lacto and neolacto series	24	1	1.73995698	1.134569	0.13605017	0.14332003
Secondary bile acid biosynthesis	1	1	1.85616217	1.13606051	0.13775027	0.14401164
Terpenoid backbone biosynthesis	23	20	1.72696922	1.13615187	0.1473256	0.15286416
Biosynthesis of unsaturated fatty acids	9	5	1.7317207	1.13611707	0.16383328	0.16872383
D-Arginine and D-ornithine metabolism	2	2	1.6073305	1.1361439	0.20445917	0.2090027
Polyketide sugar unit biosynthesis	4	4	1.55541929	1.13614467	0.21729733	0.22049288
N-Glycan biosynthesis	42	7	1.39351394	1.13579753	0.27576846	0.27778137
Synthesis and degradation of ketone bodies	5	5	0.88118043	1.13613234	0.58481416	0.58481416
